# The Portuguese man-of-war genome reveals the genetic basis of medusa loss and sea–air interface adaptation

**DOI:** 10.1093/nsr/nwag374

**Published:** 2026-06-16

**Authors:** Fanghan Wang, Yongxue Li, Yali Liu, Xiaoyun Huang, Lei Wang, Zhangyi Yu, Jiajie She, Xiaoran Ma, Qishui Qin, Wenhui Wang, Saijun Peng, Tingting Sun, Jianmin Zhao, Qiang Lin, Zhijun Dong

**Affiliations:** Muping Coastal Environment Research Station, Yantai Institute of Coastal Zone Research, Chinese Academy of Sciences, Yantai 264003, China; College of Resources and Environment, University of Chinese Academy of Sciences, Beijing 100101, China; Muping Coastal Environment Research Station, Yantai Institute of Coastal Zone Research, Chinese Academy of Sciences, Yantai 264003, China; College of Resources and Environment, University of Chinese Academy of Sciences, Beijing 100101, China; College of Resources and Environment, University of Chinese Academy of Sciences, Beijing 100101, China; State Key Laboratory of Tropical Oceanography, South China Sea Institute of Oceanology, Chinese Academy of Sciences, Guangzhou 510301, China; Guangdong Provincial Key Laboratory of Applied Marine Biology, South China Sea Institute of Oceanology, Chinese Academy of Sciences, Guangzhou 510301, China; Muping Coastal Environment Research Station, Yantai Institute of Coastal Zone Research, Chinese Academy of Sciences, Yantai 264003, China; College of Resources and Environment, University of Chinese Academy of Sciences, Beijing 100101, China; Muping Coastal Environment Research Station, Yantai Institute of Coastal Zone Research, Chinese Academy of Sciences, Yantai 264003, China; College of Resources and Environment, University of Chinese Academy of Sciences, Beijing 100101, China; College of Bio-Medicine and Health, Huazhong Agricultural University, Wuhan 430070, China; Department of Ocean Science, The Hong Kong University of Science and Technology, Hong Kong SAR 999077, China; Muping Coastal Environment Research Station, Yantai Institute of Coastal Zone Research, Chinese Academy of Sciences, Yantai 264003, China; Muping Coastal Environment Research Station, Yantai Institute of Coastal Zone Research, Chinese Academy of Sciences, Yantai 264003, China; Muping Coastal Environment Research Station, Yantai Institute of Coastal Zone Research, Chinese Academy of Sciences, Yantai 264003, China; College of Resources and Environment, University of Chinese Academy of Sciences, Beijing 100101, China; Muping Coastal Environment Research Station, Yantai Institute of Coastal Zone Research, Chinese Academy of Sciences, Yantai 264003, China; College of Resources and Environment, University of Chinese Academy of Sciences, Beijing 100101, China; Muping Coastal Environment Research Station, Yantai Institute of Coastal Zone Research, Chinese Academy of Sciences, Yantai 264003, China; College of Resources and Environment, University of Chinese Academy of Sciences, Beijing 100101, China; Muping Coastal Environment Research Station, Yantai Institute of Coastal Zone Research, Chinese Academy of Sciences, Yantai 264003, China; College of Resources and Environment, University of Chinese Academy of Sciences, Beijing 100101, China; College of Resources and Environment, University of Chinese Academy of Sciences, Beijing 100101, China; State Key Laboratory of Tropical Oceanography, South China Sea Institute of Oceanology, Chinese Academy of Sciences, Guangzhou 510301, China; Guangdong Provincial Key Laboratory of Applied Marine Biology, South China Sea Institute of Oceanology, Chinese Academy of Sciences, Guangzhou 510301, China; Muping Coastal Environment Research Station, Yantai Institute of Coastal Zone Research, Chinese Academy of Sciences, Yantai 264003, China; College of Resources and Environment, University of Chinese Academy of Sciences, Beijing 100101, China

**Keywords:** Portuguese man-of-war, genomics, single-cell transcriptome, sea–air interface adaptation, toxin

## Abstract

The most conspicuous Portuguese man-of-war, *Physalia* sp., is a neustonic hydromedusa that lacks a free-living medusa stage and features a gas-filled float and venomous tentacles. However, the molecular basis of its specialized niche adaptation remains unknown. Herein, we generated chromosome-level genomes of two neustonic hydromedusae, *Physalia utriculus* and *Porpita porpita* (with pelagic medusae). Extensive contractions of neural and muscular gene families were observed for *P. utriculus*. Loss of *Hox1* was correlated with the absence of free-living medusae, and *Hox1* knockdown caused abnormal medusa development, suggesting that *Hox1* is a key regulator of medusa development in medusazoans. Since it has lost the free-swimming medusa stage, *P. utriculus* relies on its gas-filled float and toxic tentacles to occupy its ecological niche. Single-cell transcriptomics revealed the mechanism of carbon monoxide production for float inflation. Neurotoxic, cytotoxic, and hemolytic metabolites and toxins were detected in tentacles, and the expanded *CtrA* gene with toxin domains may enhance its predation and survival. These findings reveal the genetic and cellular adaptations of *Physalia* to the sea–air interface, elucidating the crucial role that key evolutionary innovations play in shaping unique survival strategies in dynamic marine environments.

## INTRODUCTION

**Figure 1.**
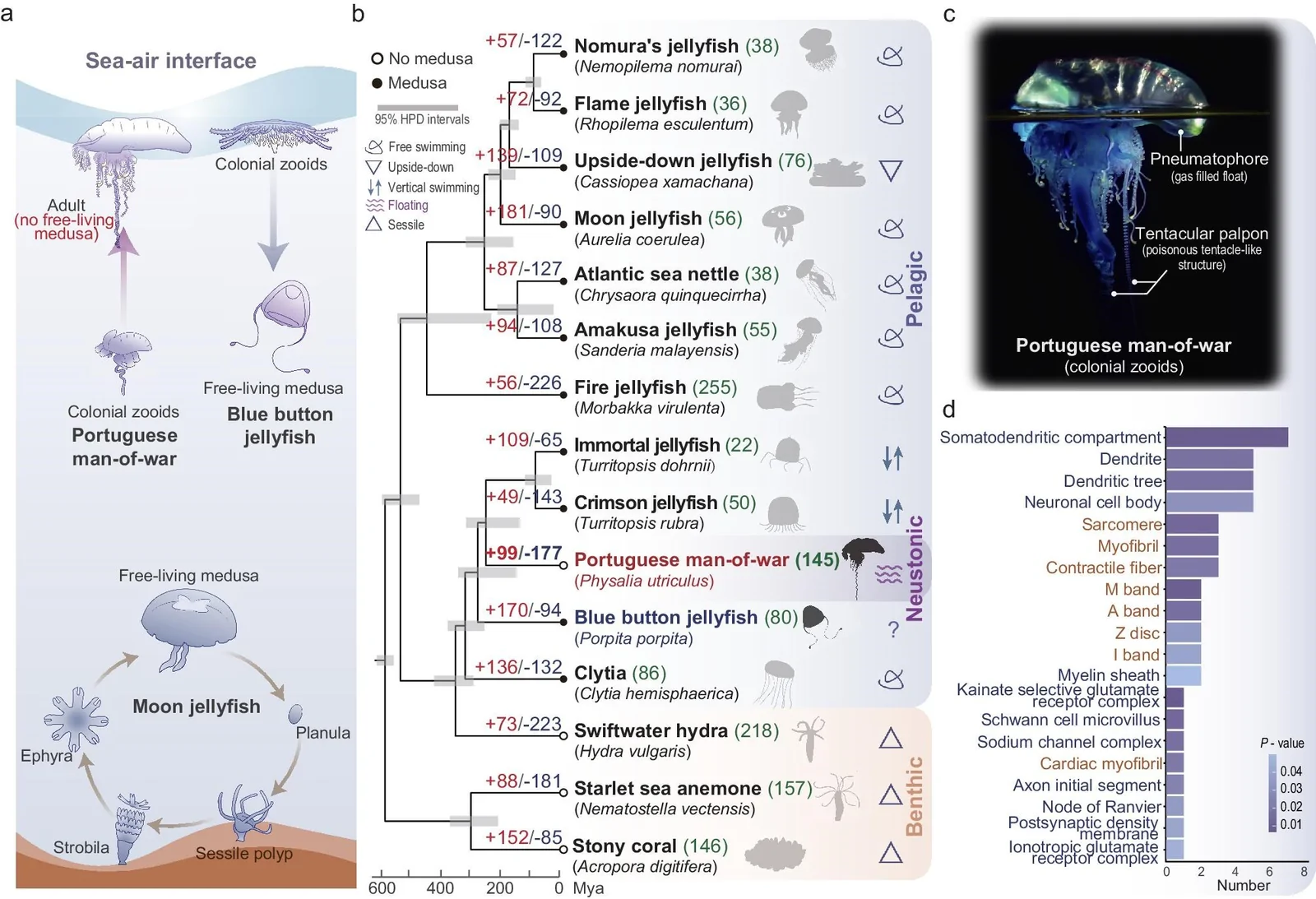
Evolution and habitat of the Portuguese man-of-war. (a) Life cycles of floating *Physalia utriculus* (neustonic colonies lacking free-living medusae), holoplanktonic *Porpita porpita* (neustonic colonies and pelagic medusa), and meroplanktonic *Aurelia coerulea* (benthic polyp and pelagic medusa). (b) Phylogenetic relationships and habitat evolution of 15 cnidarians. The numbers near each branch indicate the counts of significantly expanded and contracted gene families; the number of lost gene families is listed after each species’ name. Cnidarians exhibit a stepwise transition from benthic to neustonic and pelagic habits, with corresponding swimming patterns ranging from sessile to floating and free-swimming. The question mark indicates that the movement pattern of the medusa of *P. porpita* has not yet been recorded. (c) Morphological images of *P. utriculus* and its notable biological characteristics. (d) Gene Ontology enrichment analysis of lost gene families in the *P. utriculus* genome, focusing on the Cellular Component category. Neural-related and muscle-related functional categories are significantly enriched. The enrichment was conducted using the GOseq R package, and corrected *P* < 0.05 indicated significant enrichment.

As the closest sister group to bilaterians, cnidarians (including Anthozoa and Medusozoa) are globally distributed across shallow-to-deep waters, occupying benthic, pelagic, and neustonic habitats [1,2]. The conspicuous Portuguese man-of-war (*Physalia* sp.), a globally recognized inhabitant of the sea–air interface [3,4], belongs to the Hydrozoa (Cnidaria, Medusozoa) and represents a distinct evolutionary lineage in the diversification of animal movement patterns [5,6]. Unlike most medusozoans that use active swimming via bells, *Physalia* sp. relies on wind and currents for dispersal (Fig. 1a). Specifically, *Physalia* sp. retains the conserved colonial body plan, an ancestral trait across Hydrozoa, together with lineage-specific specializations that have evolved, making it invaluable for comparative studies on animal multicellularity and functional differentiation [7,8]. It lacks a free-living medusa stage, a hallmark of most medusozoans [4]. Instead, it develops a gas-filled float (pneumatophore) that suspends specialized zooids dedicated to feeding, reproduction, and defense [7]. Notably, its tentacular palpons contain potent toxins, among the most virulent in cnidarians, enabling efficient prey capture and deterrence [7,9,10]. Despite these unique traits, the genetic and evolutionary mechanisms underlying their origin remain poorly understood, limiting our understanding of the adaptive success of *Physalia* sp. Elucidating these mysteries could transform our knowledge of the evolution of animal locomotor systems and the genetic foundation of phenotypic innovation in marine organisms.

Most medusazoans exhibit a metagenetic life cycle characterized by alternation between sessile asexual polyps and free-swimming sexual medusa stages [11]. Evolution of the medusa stage represents a key adaptive transition, enabling the shift from benthic sessility to pelagic mobility (Fig. 1b). Free-swimming medusae require specialized cellular and organ systems for active locomotion, such as motor neurons and striated muscles [12,13]. However, the reduction or absence of the free-swimming medusa stage is observed in many lineages across the Hydrozoa, potentially representing an adaptive response to environmental constraints [14]. Although the emergence of the medusa stage has been a focal point in evolutionary biology, the genetic regulatory mechanisms governing its development remain poorly understood. Homeobox genes exert a substantial influence on the evolution of segmental development in invertebrates [15], and exhibit distinct stage-specific expression patterns during the ontogenesis of certain medusozoans, leading to the hypothesis that they serve as key molecular switches in medusa development [11,16]. Lacking the active, motile medusa stage, *Physalia* sp. colonies utilize a gas-filled pneumatophore for neustonic buoyancy (Fig. 1c), which may reflect its specialized adaptation to the sea–air interface. This structure is absent in medusa-dominated lineages [17], making *Physalia* sp. an ideal model system for investigating the genetic basis of the absence of free-living medusae. However, prior studies have predominantly described spatiotemporal expression profiles of homeobox genes [11,13,16], leaving the functional mechanisms underlying their role in medusa formation unclear.

*Physalia* sp. relies on its pneumatophore to utilize wind and oceanic currents, facilitating long-distance dispersal across tropical and subtropical marine ecosystems [8]. The pneumatophore contains carbon monoxide (CO) comprising 0.5%–13% of its gas content, which is synthesized by a gas gland using L-serine metabolism to inflate the float and maintain surface buoyancy [18]. CO secretion may inflate the float of *Physalia* sp., enabling juveniles to ascend to the ocean surface [19]. In adults, CO within the pneumatophore is gradually replaced by air through diffusion at the sea–air interface, supporting wind-driven dispersal over vast distances [18]. This adaptive strategy of *Physalia* sp. underscores its broad distribution and capacity to colonize new habitats [8]. Despite lacking free-living medusae for active locomotion, *Physalia* sp. achieves ecological success supported by its exceptionally elongated tentacular palpons (up to 30 m in length) that are armed with specialized stenotele nematocysts. These structures feature helical tubules optimized for piercing fish scales and injecting complex venom cocktails, serving pivotal roles in predation and defense [9]. The venom of *Physalia* sp. also poses a substantial risk to humans; stranded colonies on beaches can inflict severe stings via nematocysts that remain active postmortem, threatening coastal fisheries and tourism industries [10]. Despite previous efforts to identify specific venom components, including hemolytic proteins, phospholipases, serine proteases, hydrolases, and metalloendoproteases that disrupt cellular membranes and neuromuscular function, thereby rapidly immobilizing prey [20–22], a comprehensive characterization of *Physalia* sp. toxins remains incomplete. In addition, the genetic, cellular, and evolutionary processes governing the development and evolution of these distinct traits, such as the CO-related pneumatophore and venomous tentacular palpons, have not been investigated. Deciphering these processes is crucial for uncovering the genetic determinants and evolutionary innovations that drive the diversification of cnidarians.

Studies on *Physalia* sp. have been constrained by their fragile colonial structure and challenges associated with natural collection, limiting insights into their adaptations to the sea–air interface [7]. Existing genomic research has focused on genome size [23], species delineation [8], and phylogeny [24]; however, the molecular mechanisms underlying their specialization in the neustonic environment remain poorly understood. In this study, we integrated chromosome-level genome assemblies of two neustonic hydromedusae, *Physalia utriculus* (neustonic colonies lacking free-living medusae) and *Porpita porpita* (neustonic colonies with free-living medusae), with single-cell transcriptomics, metabolomics, and proteomics analyses. We reveal that Hox1 transcription factor loss in *P. utriculus* is associated with the absence of free-living medusae. The cellular and molecular mechanisms of CO production and expansion of toxin-related genes in *P. utriculus* were further investigated. The study findings illustrate the genetic basis of *P. utriculus*’s adaptation to the sea–air interface and its ecological success, highlighting how genomic innovations drive specialized survival strategies in dynamic marine environments.

## RESULTS

### Hydrozoan genomic features and genetic adaptations to the sea–air interface

Using PacBio sequencing technology, we generated two chromosome-level reference genomes for the hydromedusae, *P. utriculus* (2*n* = 20) and *P. porpita* (2*n* = 14), spanning 3.49 Gb and 4.53 Gb and containing 29 087 and 34 914 protein-coding genes, respectively, consistent with previous karyotype observation and the public genome of *P. physalis* [24,25]. With contig N50 of 1.90 Mb and 3.78 Mb and high Benchmarking Universal Single-Copy Orthologs (BUSCO) completeness (95.68% and 96.07%), respectively, these assemblies represent high-quality genomic resources.

Genomes of 13 published cnidarians and the newly sequenced *P. utriculus* and *P. porpita*, which have evolved a neustonic habit and floating behavior (Fig. 1b), were compared against those of cnidarians from divergent classes (ranging from sessile anthozoans to free-swimming scyphozoans). Phylogenomic analysis assigned *P. utriculus* and *P. porpita* to a position within the Hydrozoa. Overall, 177 significantly contracted and 145 lost gene families were identified in the *P. utriculus* genome (Datasets S1 and S2). GO enrichment of the lost gene families highlighted signatures associated with neural and muscular systems, including the somatodendritic compartment [26], glutamate receptor [27], sarcomere, and myofibril (Fig. 1d and Dataset S3) [28], as corroborated through analyses of the contracted genes (Fig. S4 and Dataset S4). These contracted or lost gene families have substantially contributed to modifications identified in the neural and muscular systems of cnidarians. They are presumably linked to the modifications of locomotor structures and sensory functions in colonial organisms (Fig. S5). Together with the positively selected genes (PSGs) and rapidly evolving genes (REGs) related to structure, nervous, and muscle system development, these changes likely drove the evolutionary absence of a free-living medusa stage in *P. utriculus* and the specialization of its floating behavior (Fig. S6, Datasets S5 and S6). The loss of adhesive-related genes (*Fbxl6, SraP*, and *Ptpn12*) in both *P. utriculus* and *P. porpita* may have also promoted their divergence from sessile lifestyles and driven the evolution of a neustonic survival strategy [29].

### Cellular and molecular characteristics involved in the absence of free-living medusae of *P. utriculus*

**Figure 2.**
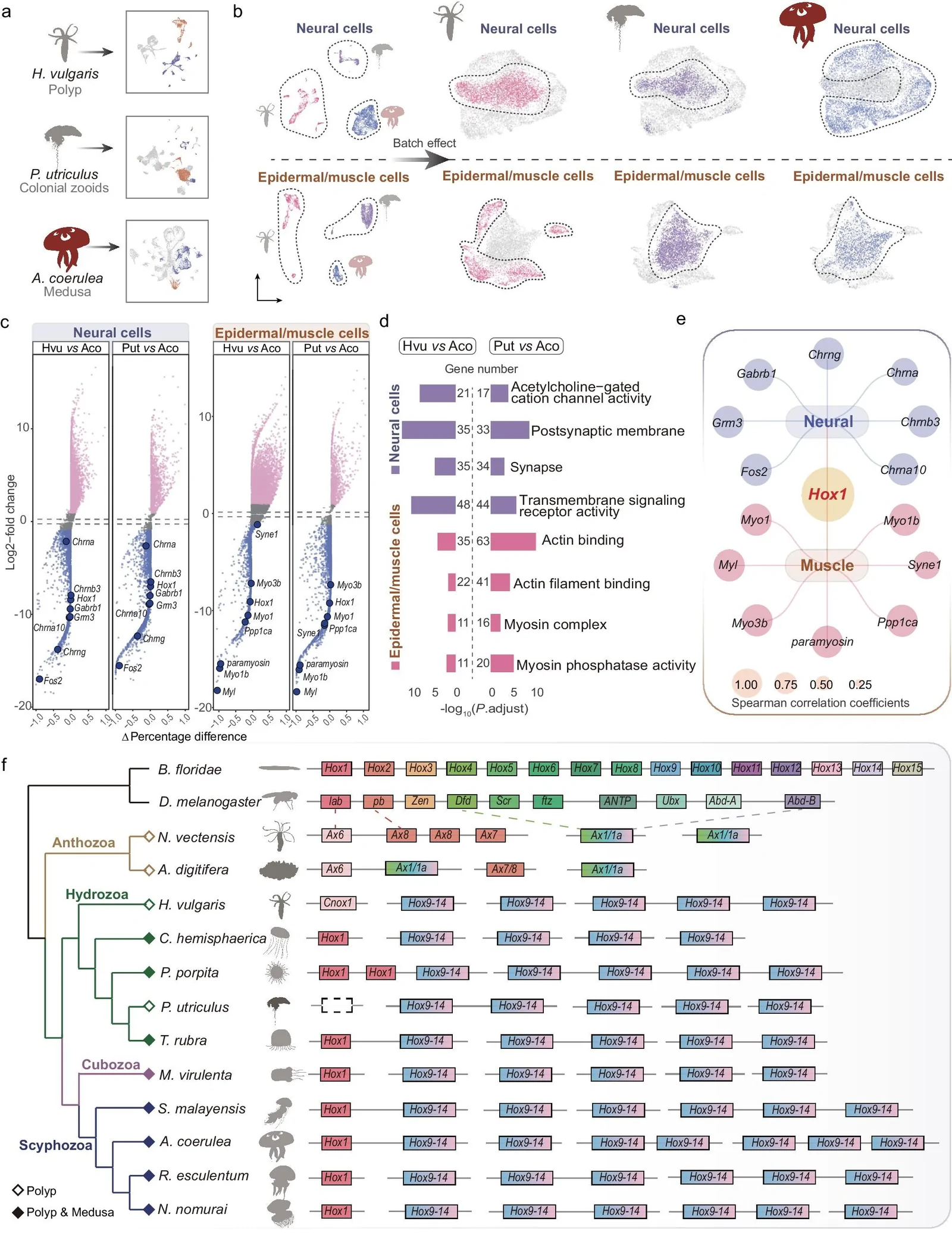
Cross-species comparison reveals the importance of the *Hox1* gene in the medusa stage. (a) Schematic diagram illustrating the life stages and body plans of *Hydra vulgaris* (polyp), *Physalia utriculus* (colonial zooids), and *Aurelia coerulea* (medusa), alongside representative uniform manifold approximation and projection plots showing neural and epidermal/muscle cell clusters. (b) Canonical correlation analysis-based integration of neural (top) and epidermal/muscle (bottom) cell populations across the three species. Dashed lines indicate cluster boundaries. (c) Differential gene expression analysis between *A. coerulea* and the two polyp-stage species (*H. vulgaris* and *P. utriculus*) for neural (left) and epidermal/muscle (right) cell groups. Abbreviations: Hvu, *H. vulgaris*; Aco, *A. coerulea*; Put, *P. utriculus.* (d) Gene Ontology enrichment analysis of differentially expressed genes in neural and epidermal/muscle cells, grouped by species comparison. (e) Co-expression analysis of *Hox1* with neural- and muscle-related genes in *A. coerulea*. Circle size represents correlation strength. Spearman correlation coefficients were used to quantify the strength of expression correlations, with circle size corresponding to the absolute value of the correlation coefficient. (f) Genomic organization of *Hox* genes in cnidarians in a phylogenetic context. Different homologous genes are labeled with colored boxes; black dashed boxes represent the absence of genes orthologous to *Hox1*.

Neural and muscle cells are essential for medusa development and swimming locomotion in cnidarians, underpinning the morphogenesis and functional manifestation of the medusa body [30,31]. To investigate the cellular composition and molecular characteristics of the neural and muscular systems during the medusa stage, single-cell transcriptome sequencing was conducted on a complete colony of *P. utriculus*. We focused on the identification of neural and epidermal/muscle cells in *P. utriculus*. These cell-type annotations were then integrated with published datasets from two reference species, *Hydra vulgaris* [32], which lacks a medusa stage, and *Aurelia coerulea* [12], which exhibits a medusa stage (Fig. 2a and b). After batch effect correction using canonical correlation analysis, expression conservation across species varied by cell type: neural cells from *H. vulgaris* and *P. utriculus* were more similar to each other, whereas epidermal/muscle cells were more similar in *P. utriculus* and *A. coerulea* (Fig. 2b).

An integrated analysis of gene expression and genomic information further revealed medusa stage-specific specialization in the neural and muscular systems. Several neural genes that were predicted to be lost or contracted in the genomes of *H. vulgaris* and *P. utriculus*, including *Grin, Cacna1, Gpr61*, and *gur-3*, were only expressed in the *A. coerulea* neural cell groups (Fig. S9). However, the muscle development-related PSGs, *Pomgnt2* and *Rnf14*, were absent in *A. coerulea* epidermal/muscle cells but retained in both *H. vulgaris* and *P. utriculus* (Fig. S10). Differential gene expression analysis indicated the molecular basis of these differences (Fig. 2c and Dataset S7). In neural cell groups, genes associated with neuromuscular junction function, including nicotinic acetylcholine receptor subunits (*Chrna, Chrna10, Chrnb3*, and *Chrng*) [33] and synaptic activity regulators (*Gabbr1* and *Grm3*) [34,35], were upregulated in *A. coerulea* but either weakly expressed or undetectable in *H. vulgaris* and *P. utriculus*. In epidermal/muscle cells, genes involved in muscle contraction (such as *Myo1, Myo3b, Myl, paramyosin*, and *Myo1b*) and the phosphatase, *Ppp1ca*, which modulates myosin phosphorylation [36,37], were also highly expressed in *A. coerulea*. GO enrichment analysis supported these observed differences (Fig. 2d and Dataset S8). In *A. coerulea* neural cells, the enriched GO terms were primarily associated with synaptic signaling and neurotransmission, including acetylcholine-gated cation channel activity, synapse, and transmembrane signaling receptor activity. The enriched GO terms in epidermal/muscle cells included actin binding, myosin complex, and muscle-associated phosphatase activity, all of which are functionally related to contractile dynamics.

Genomic comparisons indicated that the transcription factor, *Hox1*, was absent from the genomes of *P. utriculus* but specifically expressed in both the neural and epidermal/muscle cells of *A. coerulea* (Fig. 2c, Figs S9 and S10). Correlation analysis further demonstrated strong co-expression between *Hox1* and several key differentially expressed genes (DEGs) involved in neural and muscle function (Fig. 2e), suggesting that *Hox1* may play a role in regulating neural and epidermal/muscle cell fate during medusa stage development.

To further assess the evolutionary conservation of *Hox1* and its relationship with medusa formation, a comparative analysis of the *HoxL* gene class was conducted across representative cnidarian taxa. The common cnidarian ancestor likely had a *Hox* cluster consisting of anterior (*Hox1* and *Hox2*) and central/posterior (*Hox9-14*) class genes (Fig. 2f) [38]. *Cnox1* of *H. vulgaris* and *Ax6* of anthozoans have been recognized as the putative orthologs of *Hox1*; however, neither the gene order nor content was conserved between these two cnidarian groups [16], suggesting potential functional differences for *Ax6*. The orthologous gene, *Cnox1*, in *H. vulgaris* is located in the outgroup of other medusozoan *Hox1* genes (Fig. S11). This specialization of the *Cnox1* sequences, along with the differences in conserved homeodomain sequences and predicted protein structure, may lead to functional changes when compared with *Hox1* (Fig. S12). No putative orthologs of *Hox1* were found in *P. utriculus*, indicating that it may be associated with emergence of the medusa stage in cnidarians.

### *Hox1* is a key regulator of medusa formation in medusozoans

**Figure 3.**
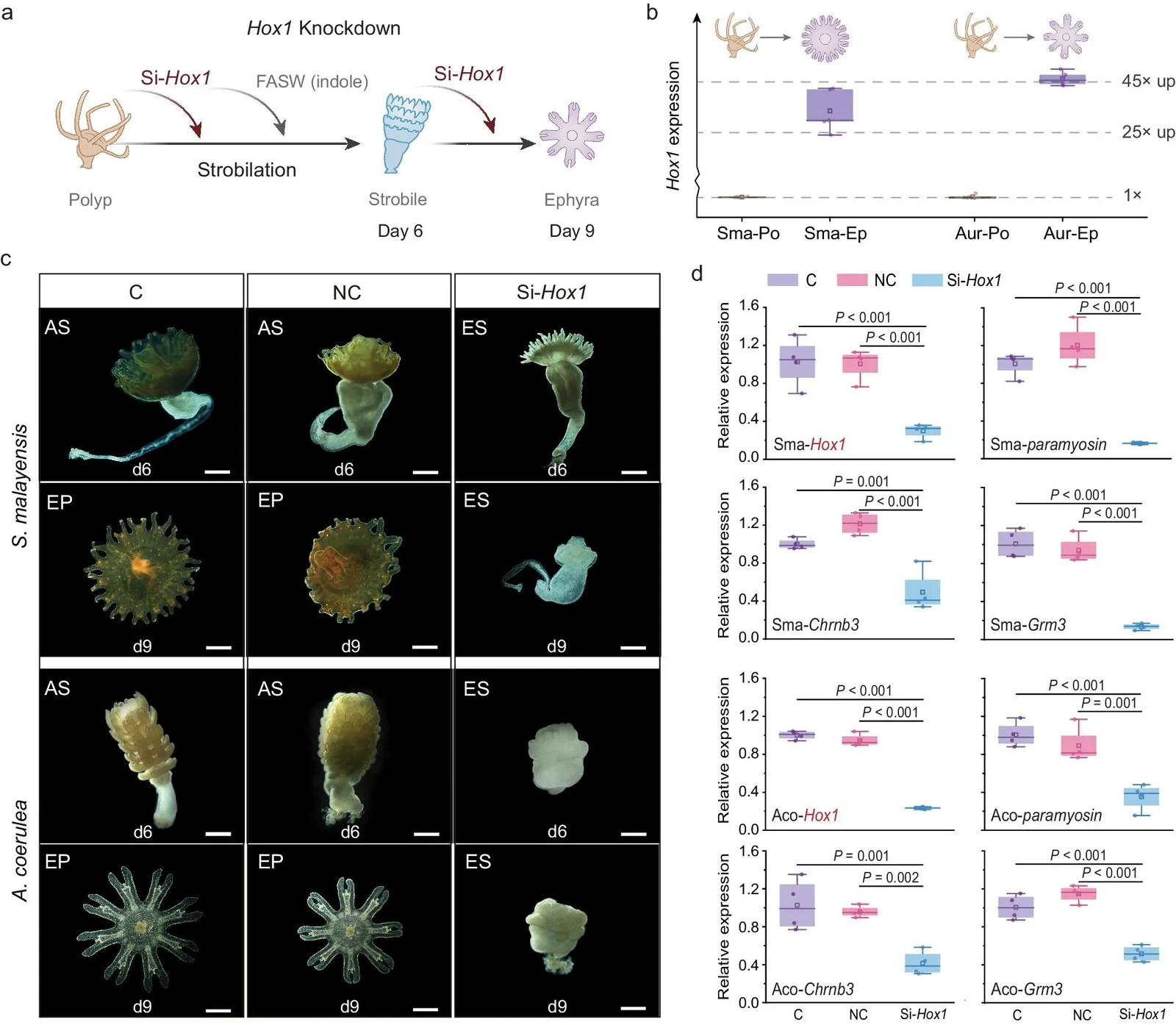
Expression and function of the *Hox1* gene in medusae. (a) Schematic of the RNA interference experiment. (b) *Hox1* gene expression in two different developmental stages of *Sanderia malayensis* and *Aurelia coerulea*. (c) Morphology of *S. malayensis* and *A. coerulea* polyps on days 6 and 9 after *Hox1* knockdown. Abbreviations: AS, advanced stage of strobilation with long lappets and well-developed rhopalia; ES, early stage of strobilation with undegenerated tentacles and newly formed segments; EP, ephyra stage. Scale bar: 500 μm. (d) Relative expression of target genes in the si-*Hox1* (*Hox1* siRNA interference) groups.

The polyp-to-medusa transition represents a crucial shift in the life strategies of certain cnidarians that exhibit a medusa stage. Among scyphozoans (often referred to as “true jellyfish”), newly released ephyrae (EP) from strobilating polyps are considered juvenile medusae and exhibit fundamental medusa traits [39]. *Hox1* expression was detected before and after the transition of two typical species: the single-ephyra strobilating *Sanderia malayensis* and multi-ephyrae strobilating *A. coerulea. Hox1* was barely detectable in polyp samples, with expression markedly elevated in the EP stage (Fig. 3b), indicating that this gene is preferentially expressed in the medusa stage and may be related to regulation of the morphological development of medusa.

To investigate the functional impact of *Hox1* on medusa formation, we knocked down *Hox1* genes during the strobilation process of *S. malayensis* and *A. coerulea* using short interfering RNA (siRNA) against *Hox1* (Fig. 3a). After 9 days of incubation with the transfection complex that was replaced every 3 days, polyp morphology in the si-*Hox1* (*Hox1* siRNA interference) group was substantially different from that in the other treatment groups. In the control (C) and siRNA negative control (NC) groups, nearly all polyps of *S. malayensis* and *A. coerulea* formed segments and developed into an advanced stage of strobilation on day 6, with long lappets and well-developed rhopalia, and EPs were released on day 9. In contrast, all polyps of *A. coerulea* and most of *S. malayensis* in the si-*Hox1* group remained in the early stage of strobilation, with undegenerated tentacles and newly formed segments. EP failed to develop normally until day 9 (Fig. 3c and Table S15). Quantitative reverse transcription PCR (RT-qPCR) revealed that the si-*Hox1* group exhibited significant downregulation of the *Hox1* gene compared with that in the control and NC groups, confirming the effectiveness of the siRNA used (*P* < 0.001). The expression of genes related to muscle and nerve development (such as *paramyosin, Chrnb3*, and *Grm3*) was significantly downregulated in the si-*Hox1* groups (Fig. 3d).

### Cellular and molecular mechanisms of carbon monoxide production in *P. utriculus*

**Figure 4.**
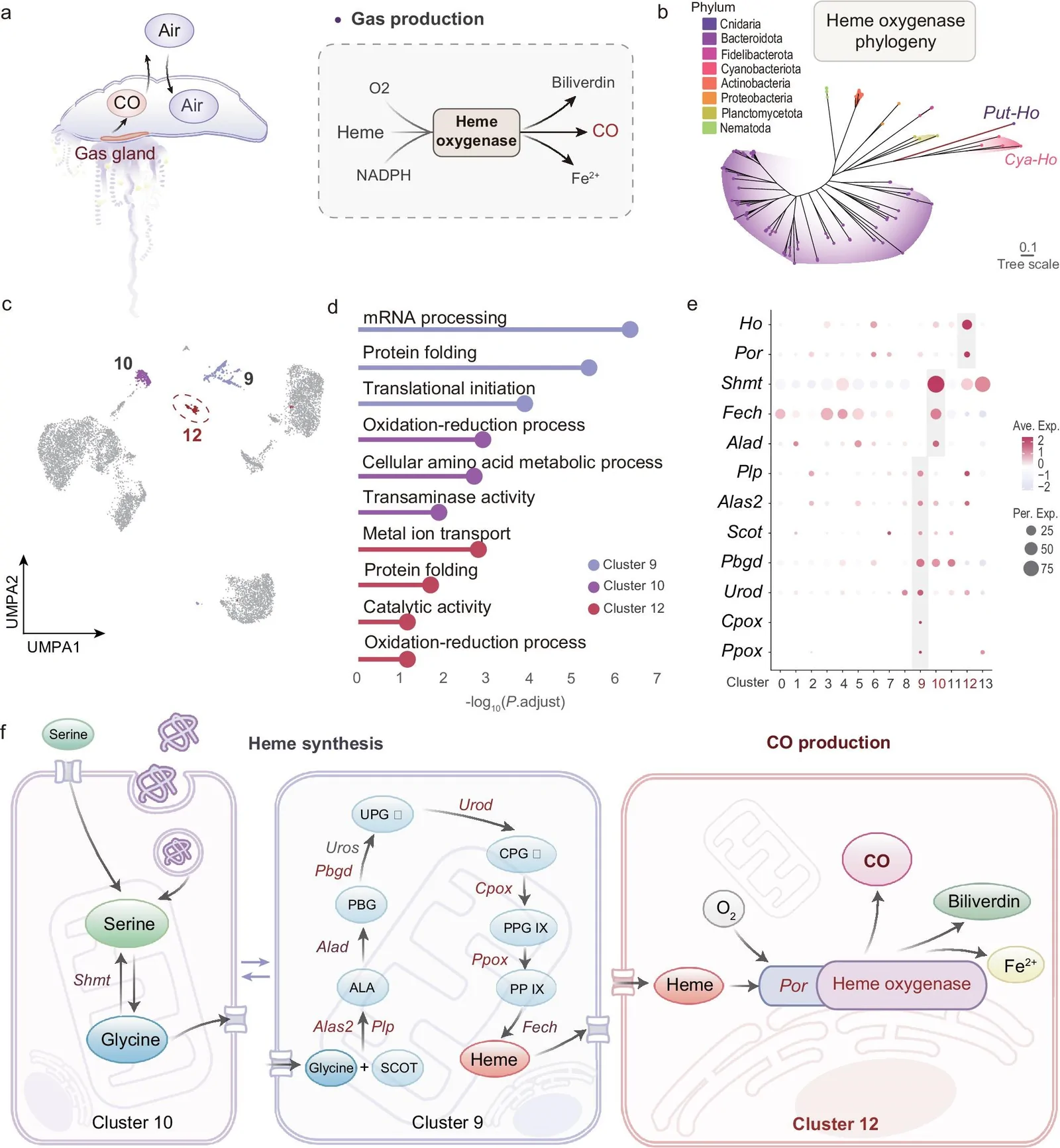
Characterization of gas production in *Physalia utriculu*s. (a) The gas gland in the pneumatophore of *P. utriculus* produces carbon monoxide (CO) via a heme oxygenase-mediated mechanism, which enables floating. (b) Phylogeny of the *P. utriculus* heme oxygenase gene, involving horizontal gene transfer, as a sister group to bacterial homologues. (c) Uniform manifold approximation and projection plot of the pneumatophore highlighting clusters 9, 10, and 12, which are associated with distinct modules of the CO biosynthetic pathway. (d) Gene Ontology enrichment analysis of clusters 9, 10, and 12. (e) Dot plot showing the expression of genes involved in the heme biosynthesis and CO production pathways across cell clusters in the *P. utriculus* pneumatophore. (f) Schematic diagram of the metabolic pathways involved in heme synthesis and CO production in different clusters. Cluster 10 supplies heme precursors and encodes key rate-limiting enzymes; cluster 9 cooperates with cluster 10 to synthesize heme; and cluster 12 catalyzes heme degradation to produce CO.

The ability to float and sail at the ocean surface is a key trait of *P. utriculus* that is supported by its complex pneumatophore structure. The gas gland, a specialized ectodermal tissue, secretes gases into the lumen of the float, creating a buoyant organ that can be tilted via muscular contractions to catch wind [25]. The *P. utriculus* floats at the sea–air interface by producing CO via its gas gland [7], a process that relies on the heme oxygenase gene (Fig. 4a) [18,40]. Phylogenetic analysis showed that the heme oxygenase gene in *P. utriculus* (*Put-Ho*) formed a sister-group relationship with those from the Candidatus Marinamargulisbacteria bacterium (Fig. 4b). As widely used indicators of horizontal gene transfer (HGT), an Alien Index (AI) value of 8 and Out_pct of 97.33% support horizontal acquisition from bacteria [41]. The gene likely originated in bacteria and was transferred to an ancestor of *P. utriculus*. This may have enabled the evolution of CO-dependent buoyancy control—a trait not universally present in related cnidarians.

To investigate the cellular basis of CO production in *P. utriculus*, single-cell transcriptome sequencing was specifically conducted on the pneumatophore, which houses the gas gland tissues (Fig. S13). Unsupervised clustering revealed that clusters 9, 10, and 12 exhibited high expression of key genes involved in the CO production pathway [42,43], including enzymes required for heme biosynthesis and degradation (Fig. 4c–e). Cluster 12 showed specific enrichment of *Put-Ho*, the terminal enzyme catalyzing CO generation from heme [40]. GO enrichment analysis further supported these findings, showing that cluster 12 was significantly enriched in oxidation-reduction processes, catalytic activity, and protein folding (Fig. 4e), consistent with its role in heme catabolism and CO production. Clusters 9 and 10 cooperatively participate in heme biosynthesis. Cluster 9 was enriched in genes associated with mRNA processing and protein folding, indicating its central role in executing core heme synthetic steps [44]. The initiating *Alad* and terminal *Fech*, together with upstream *Shmt*, were predominantly enriched in cluster 10 (Fig. 4e), which was characterized by an enrichment of genes involved in amino acid metabolism, serving as essential precursors in the metabolic network related to heme synthesis and, by extension, CO production [45]. Functional diagrammatic integration of cluster-specific gene expression revealed a three-cluster division of labor: cluster 10 contributes to glycine‑serine metabolism and provides rate‑limiting enzymes for heme synthesis, cluster 9 executes the core heme biosynthetic reactions in synergy with cluster 10, and cluster 12 executes the terminal heme degradation step, leading to CO production (Fig. 4f). These findings suggest that CO biosynthesis in the gas gland is coordinated through distinct metabolic modules distributed across multiple specialized cell types.

### Multi-omics analysis revealed the toxin and venom characteristics in *P. utriculus*

**Figure 5.**
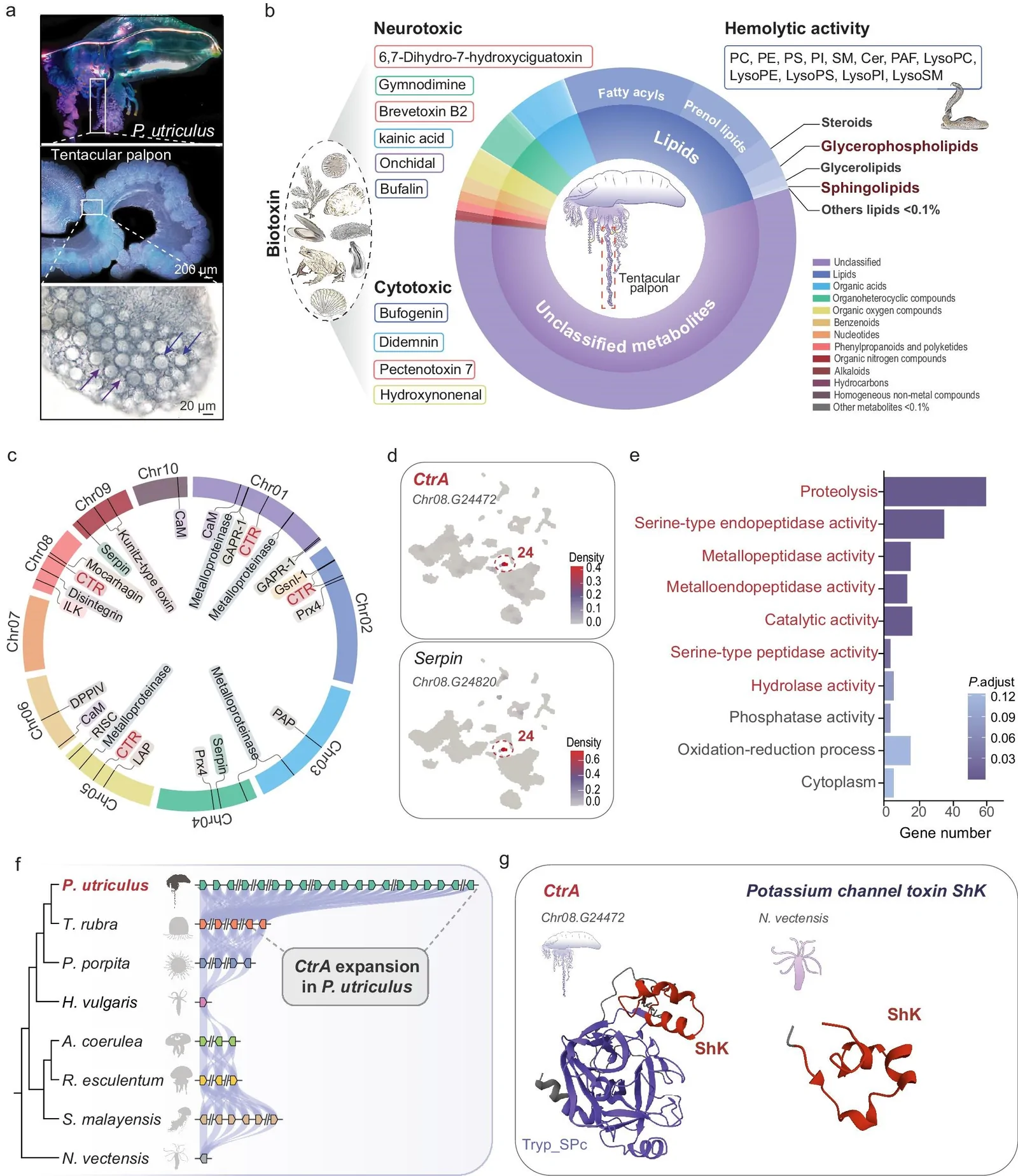
Characteristics of *Physalia utriculus* toxins. (a) Images of *P. utriculus* tentacular palpons and nematocysts, with different colored arrows indicating two types of nematocysts. (b) Metabolome analysis of *P. utriculus* tentacular palpons reveals the presence of neurotoxins, cytotoxins, and hemolytic metabolites. (c) Proteome analysis of *P. utriculus* tentacular palpons reveals the chromosomal distribution of toxin proteins. (d) Feature plots show toxin-related genes (*CtrA* and *Serpin*) expressed in *P. utriculus*, with high expression observed in cluster 24. (e) Gene Ontology enrichment analysis of genes specifically upregulated in cluster 24. (f) Expansion of *CtrA* in the *P. utriculus* genome. (g) *CtrA* protein structure prediction and its structural similarity to anemone toxins.

Understanding the toxicity of *P. utriculus* is of paramount importance, given its notable ecological role and potential threats to human health and other organisms. The tentacles of *P. utriculus* are distinct from those of many jellyfish in that two distinct sizes of nematocysts are located on only one side of the tentacular palpons (Fig. 5a). To investigate the composition of molecules with potential venom-related activity in *P. utriculus*, a comprehensive metabolomic analysis of the tentacular palpons was conducted to identify metabolites previously reported to exhibit toxic properties in other organisms. Using the liquid chromatography (LC)-quadrupole time-of-flight platform, qualitative and quantitative analyses were conducted on six replicated samples. In total, 4268 metabolites were annotated. By focusing on metabolites with a mass error (in ppm) set to an absolute value <10 [46], we found that lipids formed the highest proportion of the classified metabolites at 26.1%. Glycerophospholipids (3.3%) and sphingolipids (0.7%), which are known for their hemolytic activity and are major lipid components in snake venoms, were detected [47]. Various neurotoxic or cytotoxic metabolites, including bufalin, bufogenin, gymnodimine, microcystin, brevetoxin B2, and pectenotoxin 7, which have been reported in toads, dinoflagellates, and other poisonous organisms [48–54], were also detected in the tentacle metabolome of *P. utriculus* (Fig. 5b). Several metabolites related to blood coagulation and platelet activation were also detected (Dataset S9), which may play a role in immobilizing prey or modulating physiological responses during predation.

The proteome of the tentacular palpons was also analyzed. Out of 746 detected proteins via the applied LC-mass spectrometry (MS)/MS workflow, 31 were identified as toxin proteins based on their sequence homology to known toxin proteins and toxin-associated functional domains. These included major toxic components, such as chymotrypsin (CTR), zinc-dependent metalloproteases, serine protease inhibitors (*Serpin*), calmodulin, peroxidases, and Kunitz-type protease inhibitors (Fig. 5c and Dataset S10), which have been identified in the venoms of various toxic organisms, such as jellyfish, venomous snakes, lionfish, tarantulas, and centipedes [20–22,55–58]. Most of the genes encoding toxin proteins were specifically and highly expressed in cluster 24, such as *CtrA* (chymotrypsinogen A), *Serpin, Pla2, Spink5* (Kazal‑type serine protease inhibitor 5 precursor), and zinc metalloproteinase-coding genes (*Nas-6, Nas-14*, and *Nas-30*) (Fig. 5d and Fig. S14). GO enrichment analysis further revealed significant enrichment in biological processes that included proteolysis, serine-type endopeptidase, serine-type peptidase, and metallopeptidase activities (Fig. 5e and Dataset S11), indicating a potential functional role in protease activity and venom-associated processes [59].

Notably, toxin proteins belonging to CTR were expanded in the *P. utriculus* genome (Fig. S5). In particular, 20 copies of *CtrA* were present in *P. utriculus*, which is substantially more than that found in jellyfish (Fig. 5f). *CtrA* was predicted to comprise the ShK domain, which has been reported as a toxin domain in sea anemones [60], as well as the Tryp_SPc domain, which has been detected in spider venom (Fig. 5g) [61]. Coupled with the specific high expression of most expanded *CtrA* copies in cluster 24 (Fig. S14), this extensive expansion of the *CtrA* gene in *P. utriculus* may endow it with enhanced venom-related functions, potentially contributing to its predation efficiency and defense capabilities. It may also reflect an evolutionary adaptation to its ecological niche, enabling it to better compete for resources and deter predators in the sea–air interface.

## DISCUSSION

The split between anthozoans and medusozoans represents a pivotal divergence in cnidarian evolution that signifies not only physical transformation but also the holistic augmentation of functionality and adaptability, paving the way for the evolution of motor and nervous systems in advanced organisms [62]. Within the Hydrozoa, this evolutionary trajectory is marked by striking ontogenetic diversity, encompassing broad variations in polyp, colony, and medusa morphologies, as well as the independent reduction or lack of the free-living medusa stage across clades [63]. The pneumatophore (gas-filled float) in *P. utriculus* is a specialized “neoformation” rather than a modified medusoid zooid, as initially proposed by 19th century scholars [64,65]. Adapted to the sea–air interface, *P. utriculus* exhibits distinct neural- and muscle-associated gene expression profiles. These transcriptional patterns are tightly linked to extensive contractions and losses of gene families in the genome and may reflect evolutionary trade-offs tied to the lack of free-living medusae and reliance on flotation. Although medusa reduction is common across many siphonophores, *Physalia* sp. is uniquely adapted to surface floating [66]. Comparative transcriptomics revealed that the neural cells of *P. utriculus* clustered more closely with the medusa-lacking *H. vulgaris* than with the medusa-bearing *A. coerulea* (Fig. 2b). This pattern indicates the retention of ancestral neural traits adapted to polypoid lifestyles, characterized by expanded *Ty3-I* retrotransposons associated with neural plasticity [67] coupled with contractions of neural receptor genes (*Grin* and *Gpr61*) and *gur-3* chemosensory loci [68,69], which are potentially linked to the distinct sensory-motor functional characteristics of *P. utriculus*’s surface-floating lifestyle, underpinning the slow and sustained buoyancy adjustments required for surface persistence. Whereas *P. utriculus* epidermal/muscle cells exhibited a greater similarity to *A. coerulea*, the absence of the striated muscle nuclear positioning gene, *Syne1*, and reduced expression of muscle contraction genes (*Myo1, Myo3b, Myl, paramyosin*, and *Myo1b*) suggest a functional compromise in which basal contractile machinery for colony coordination is retained while specialized mechanisms for independent medusa motility are lost. This combined pattern of neural and muscular transcriptomic profiles, with each component linked to the functional demands of unique surface-floating lifestyle of *P. utriculus*, supports functional adaptation rather than phylogeny-driven similarity as the key driver of transcriptomic profiles.

The transcription factor, *Hox1*, is specifically expressed in the neural and epidermal/muscle cells of *A. coerulea* but completely absent from the genome of *P. utriculus*. Our investigation suggests a potential association between the presence of *Hox1* and free-living medusa development in cnidarians, although this relationship is constrained by the scope of species sampling, consistent with considerations of other cnidarian developmental genes [11]. Orthologous *Hox1* genes are either absent or have undergone sequence variations in clades that lack a free-living medusa stage (such as the anthozoans, *H. vulgaris* and *P. utriculus*). Initial reports found that the *Hox1* gene exhibited prominent medusa stage-specific expression patterns in *A. aurita* and *Morbakka virulenta*, suggesting a potential role in regulating polyp-to-medusa transitions [16]. In the present study, *Hox1* expression patterns observed before and after strobilation in *S. malayensis* and *A. coerulea* further support its involvement in medusa-forming stages across select medusozoans. Using previous single-cell RNA-sequencing (scRNA-seq) data from different developmental stages [12], we observed *Hox1* expression restricted to the medusa stage of *Turritopsis rubra* (specifically in striated muscle cells; Fig. S15) and to the late strobila and ephyra stages of *A. coerulea* (in striated muscle and nerve cells; Figs S9 and S10). Therefore, *Hox1* is specifically expressed in the medusa-forming stage of the hydrozoans, cubozoans, and scyphozoans we had examined, despite their divergent free-living medusa-developing mechanisms [70]. Notably, our sampling of these cnidarian classes was not exhaustive, and rare non-medusa-bearing cnidarians may retain orthologs of such genes (often as pseudogenes or with sequence alterations) [11]. *Hox1* has been reported to function as a conserved marker of medusa development, exhibiting stage-specific roles in *Aurelia* striated muscle [13,16] and the *Clytia hemisphaerica* mechanosensory system [71]. In the present study, *Hox1* knockdown prevented *S. malayensis* and *A. coerulea* from generating well-developed EP and led to significant downregulation of neuromuscular-related genes (*paramyosin, Chrnb3*, and *Grm3*). As an anterior regulator, *Hox1* likely governs free-living medusa development in these two species by modulating downstream neuromuscular developmental genes; its knockdown disrupts these processes and blocks juvenile medusa development. Notably, current evidence supports a regulatory role for *Hox1* in medusa development but does not clarify whether this regulation is direct or indirect. Future mechanistic investigations are needed to address this issue. Therefore, in *P. utriculus*, loss of the *Hox1* gene may correlate with neuromuscular development, eliminating free-living medusae and driving the evolution of colony-dependent flotation, representing an adaptive trade-off for sea–air interface survival.

Colonization of the sea–air interface likely occurred through successive transitions from the seafloor, including epibiotic attachment to organisms or rafting on debris [4]. The floating *P. utriculus* and *P. porpita* both stem from an ancestor with a complex life cycle that encompassed a substrate-attaching polyp and pelagic medusa [4]. However, loss of the *Hox1* gene in *P. utriculus* correlates with the disappearance of free-living medusae, whereas *P. porpita* retained both *Hox1* and functional medusae, revealing a correlation between the presence/absence of free-living medusae and the ancestral gain/loss of *Hox1*. Owing to the limited number of species with available annotated genomic data, additional sampling of closely and distantly related species will be necessary to further validate the broader relevance of *Hox1*. In addition, the adhesive-related genes, *Fbxl6, SraP*, and *Ptpn12*, present in the freshwater cnidarian, *Hydra magnipapillata* [29], were lost in both *P. utriculus* and *P. porpita* (Fig. S5). This collective loss promoted their divergence from sessile lifestyles and drove the evolution of a neustonic survival strategy.

As a specialized structure that enables *P. utriculus* to float, the pneumatophore synthesizes CO in its gas-gland tissue using l-serine as a key substrate [18]. The CO is then secreted into the gas cavity, providing buoyancy for floating [19]. Although Munro *et al.* performed transcriptome sequencing on the pneumatophores of several siphonophores, they only identified specific genes that were significantly differentially expressed in particular zooids, primarily including *Hox* and *Wnt* genes involved in patterning cnidarian bodies within specific expression domains [22]. The molecular mechanism underlying the production of CO in the pneumatophore remains unclear. Using single-cell transcriptomics, we found that all genes in the heme pathway by which serine generates CO in animals [42,43] were expressed in *P. utriculus* pneumatophores. The gas-gland tissue likely produces CO through metabolic collaboration among three functional cell clusters. Clusters 9 and 10 work synergistically to complete heme biosynthesis, and cluster 12 catalyzes heme degradation to produce CO, forming a unique multicellular metabolic network where clusters specialize in substrate supply, intermediate synthesis, and final catalysis. Notably, the key heme oxygenase gene (*Put-Ho*) originated from bacteria via HGT (Fig. 4B), a pivotal event that endowed *P. utriculus* ancestors with CO-dependent buoyancy-regulating capabilities. This adaptation enabled *P. utriculus* to disperse globally across seas via passive drifting and consistently occupy the sea–air interface, even in the absence of a free-living, motile medusa stage.

At the sea–air interface, the floating *P. utriculus* relies on its ultra-long tentacles for passive prey capture and has limited capacity for active predator avoidance, placing high demands on rapid prey immobilization and efficient defense [9,72]. These ecological pressures have driven adaptive evolution of the venom system. Multi-omics analyses were performed on the tentacular palpons of *P. utriculus*. Metabolites are often overlooked in toxin analyses of cnidarians [20,21]. Our study identified metabolites reported to exhibit neurotoxic or cytotoxic activity in poisonous organisms [48–54], including hemolytic glycerophospholipids, sphingolipids, and blood coagulation-related metabolites also reported in snake venom [47], that collectively constituted a substantial proportion of the metabolites present in the tentacular palpons of *P. utriculus*. They may synergistically enhance membrane disruption, neuromuscular paralysis, and prey immobilization efficiency, aligning with the multi-component venom strategies of other venomous cnidarians. Toxin proteins commonly reported in toxic cnidarians, including metalloproteases, calglandulin, serine protease inhibitors, and peroxidases [20–22,73], were detected in the tentacular palpons of *P. utriculus*. Most toxin-encoding genes exhibited specific and high expression in cluster 24 of the complete colony, with GO enrichment in proteolysis and metallopeptidase activity, suggesting potential roles in protease-mediated venom functions. *CtrA* exhibited substantial genomic expansion in *P. utriculus*, containing toxin domains found in sea anemones (ShK) and poisonous spiders (Tryp_SPc) [60,61]. CTR-like genes have been isolated from the nematocyst extracts of *Nemopilema nomurai* [74] and previously associated with digestive enzyme functions in the gastrozooids of *P. utriculus* [22]. However, multiple CTR proteins were detected in the tentacular palpon proteomes. The expansion and domain fusion of these toxin genes represent a critical evolutionary strategy to enhance predation and defense capabilities, enabling *P. utriculus* to persist and compete in the dynamic, competitive sea–air interface environment.

The evolution of *P. utriculus* exemplifies cnidarian adaptation to extreme environments, which is achieved through genomic innovations, including the loss of *Hox1*, expansion of the toxin-related gene, *CtrA*, HGT for CO synthesis, and multicellular specialization in gas metabolism. This study investigated the genetic basis of ontogenetic specialization, buoyancy regulation, and toxic mechanisms through the integration of multi-omics approaches. Despite challenges in functional validation due to sample scarcity and the difficulty of performing *in situ* experiments for key gene expression, cross-species comparisons and expression profiling have provided support for associations between key genes and adaptive traits, offering new insights into cnidarian evolution and a scientific foundation for assessing coastal risks and the exploitation of marine resources. Further exploration of the regulatory networks and signaling cascades underlying the unique physiological processes of *P. utriculus* will enhance our comprehension of its chemical ecology and help elucidate its predatory strategies, defensive mechanisms, and evolutionary adaptations to the sea–air interface.

## MATERIALS AND METHODS

Adult *P. utriculus* and *P. porpita* were collected from waters near Zhaoshu Island (Hainan, China) in January 2024, identified by morphology and phylogenetic analysis (mitochondrial genome and 16S rRNA). After 2–3 days of starvation, different zooid tissues were excised for whole-genome sequencing (Illumina short-read and PacBio HiFi long-read), Hi-C sequencing, and scRNA-seq. Genomic DNA was extracted using the CTAB method.

Genome assembly was conducted using hifiasm and purge_dups, scaffolded to chromosome level with Hi-C data, and evaluated by BUSCO. Repetitive elements were annotated via homology-based and *de novo* approaches; protein-coding genes were predicted through integrated strategies (homology-based, *de novo*, transcriptome data) and functionally annotated against multiple databases. Phylogenetic analyses were performed with OrthoFinder and STAG, and divergence times estimated via MCMCTREE. Repeat landscape analysis, gene family expansion and contraction (CAFÉ), identification of positive selected and rapidly evolving genes (PAML), and synteny analysis of *CtrA* gene families were conducted.

scRNA-seq libraries were constructed using the BD Rhapsody system, processed with Seurat (v5.2.0), and cross-species comparisons performed via SAMap. Homeodomain sequence analysis involved BLAST searches and maximum-likelihood phylogenetic trees. *Hox1* gene function was verified by RNA interference and RT-qPCR. Horizontal gene transfer of the heme oxygenase gene was assessed via BLAST and phylogenetic analysis. Metabolomic (Ultra Performance Liquid Chromatography-Quadrupole Time-of-Flight Mass Spectrometry) and proteomic (mass spectrometry) analyses of tentacular palpons were conducted, with toxins identified via BLASTp against venom databases.

Detailed materials preparation and methods are available in the online Supplementary information.

## Supplementary Material

nwag374_Supplemental_Files

## Data Availability

The whole-genome assemblies of *P. utriculus* and *P. porpita* were deposited in the NCBI database under accession code PRJNA1279273, and raw reads from the scRNA-seq data of *P. utriculus* deposited under accession code PRJNA1277818. Protein MS data are publicly available at the ProteomeXchange Consortium in the PRIDE repository with the identifier PXD065591. Metabolite MS data are available from the MetaboLights repository, with reference MTBLS12736. All other data are available in the main text or the Supplementary information.
