# Supplementary material for: The Portuguese man-of-war genome reveals the genetic basis of medusa loss and sea–air interface adaptation
Source: Natl Sci Rev. 2026 Jun 16;13(17):nwag374. doi: 10.1093/nsr/nwag374 (PMC13579702; doi:10.1093/nsr/nwag374)
Supplement: Supplementary file 1 [file nwag374_supplemental_files.zip › Supplementary_Information_resubmit.docx]

**Supplementary Information to:**

**The Portuguese man-of-war genome reveals the genetic basis of medusa loss and sea–air interface adaptation**

**Authors:** Fanghan Wang^1,2,†^, Yongxue Li^1,2,†^, Yali Liu^2,3,4,†^, Xiaoyun Huang^1,2,†^, Lei Wang^1,2,†^, Zhangyi Yu^5^, Jiajie She^6^, Xiaoran Ma^1^, Qishui Qin^1^, Wenhui Wang^1,2^, Saijun Peng^1, 2^, Tingting Sun^1,2^, Jianmin Zhao^1,2^, Qiang Lin^2,3,4^*, Zhijun Dong^1,2^*

†Authors contributed equally to this work.

*Correspondence to: zjdong@yic.ac.cn (Z.J.D.); linqiang@scsio.ac.cn (Q.L.)

**Affiliations:**

^1^Muping Coastal Environment Research Station, Yantai Institute of Coastal Zone Research, Chinese Academy of Sciences, Yantai, Shandong 264003, China.

^2^College of Resources and Environment, University of Chinese Academy of Sciences, Beijing 100101, China.

^3^State Key Laboratory of Tropical Oceanography, South China Sea Institute of Oceanology, Chinese Academy of Sciences, Guangzhou 510301, China.

^4^Guangdong Provincial Key Laboratory of Applied Marine Biology, South China Sea Institute of Oceanology, Chinese Academy of Sciences, Guangzhou 510301, China.

^5^College of Bio-Medicine and Health, Huazhong Agricultural University, Wuhan, Hubei 430070, China.

^6^Department of Ocean Science, The Hong Kong University of Science and Technology, Hong Kong SAR 999077, China.

**Supplementary Information**

**Table of Contents**

**Detailed methods	Page 3-12**

**Figures S1-S15	Page 13-27**

**Tables S1-S16	Page 28-43**

**Datasets S1-S13	Page 44**

**Reference	Page 45-46**

**Detailed methods**

**Sample collection, identification, and nucleic acid sample preparation**

Adult colonies of *Physalia* sp. and *P. porpita* were collected from waters adjacent to Zhaoshu Island (Hainan Province, China) in January 2024 (Table S1). All specimens were transported to laboratory aquariums for temporary maintenance until subsequent sampling and analysis.

Recent integrative taxonomic studies of *Physalia* have resolved this cosmopolitan genus into four morphologically and genetically distinct species: *P. physalis*, *P. utriculus*, *P. megalista*, and *Physalia* sp. C2 [1]. To confirm the species identity of our *Physalia* specimens, we first targeted *P. utriculus* by screening for key morphological traits consistent with its diagnostic criteria, including a pneumatophore measuring roughly 5–8 cm in length, a single major tentacle, yellow-tipped gastrozooids, and a flared posterior growth zone. (Fig. S1a). The mitochondrial genome and 16S rRNA sequence were used for phylogenetic analysis following the methodological framework of Church et al. [1]. Sequences were aligned with MAFFT (v7.511), and a maximum-likelihood (ML) phylogenetic tree inferred using IQ-TREE (v2.3.4) with 1,000 bootstraps. Phylogenetic placement of our specimens nested within the *P. utriculus* clade (Figs. 1c and 1d), consistent with morphological observations, confirming their identity as *P. utriculus*. In addition, the other specimens were identified as *P. porpita* based on the typical morphological characteristics of the samples: a disc-shaped chitinous float with concentric rings (2–5 cm in diameter), a central conical gastrozooid on the float’s ventral surface, surrounded by smaller gastrozooids, granular gonozooids attached to the base of small gastrozooids, and marginal dactylozooids with longitudinal branches terminating in cnidocyte knobs (Fig. S1b).

Zooid samples of *P. utriculus* colonies, including the pneumatophore, tentacular palpons, gastrozooid, and gonodendron, were carefully excised from their attachments using a scalpel. The *P. utriculus* pneumatophore and intact colonial zooids (complete colony) of *P. porpita* were subjected to whole-genome sequencing. A sample of the tentacular palpons from the same colony of *P. utriculus* and same complete colony of *P. porpita* were also used for Hi-C sequencing. Before DNA extraction, the samples were starved for 2–3 days. Genomic DNA was then extracted from each sample using a standard cetyltrimethylammonium bromide extraction method [2]. Samples of the pneumatophore, tentacular palpons, gastrozooid, and gonodendron of *P. utriculus* were processed according to the manufacturer’s protocol for scRNA-seq. The mantle, dactylozooids, gastrozooid, and gonozooids of *P. porpita* were carefully dissected from complete colonies for single-cell transcriptome sequencing to assist in gene annotation.

**Genome sequencing and assembly**

Genome sequencing for both *P. utriculus* and *P. porpita* was performed by Yuda Biotechnology Co., Ltd. (Guangzhou, China) using both short-read and long-read technologies. Short-read sequencing used Illumina platforms with small fragment library construction. DNA was fragmented using ultrasonic disruption, end-repaired, A-tailed, ligated with adapters, and amplified using PCR before sequencing. Raw short-read data were filtered using SOAPnuke (v1.5.6) with parameters “-n 0.01 -l 20 -q 0.1 -i -Q 2 -G -M 2 -A 0.5 -d” to remove adaptor-containing and low-quality reads (with >10% bases of quality ≤20 or >1% N bases). We performed long-read sequencing using the PacBio Sequel platform with HiFi library preparation. DNA was fragmented using g-TUBE, repaired for damage and ends, ligated with hairpin adapters to form SMRTbell templates, and sequenced on the PacBio Sequel platform. Raw PacBio data were processed using CCS program of the SMRT Link (v9.0.0.92188) to generate high-quality HiFi reads with the following parameters: min-passes, 3; min-length, 1,000; and min-rq, 0.95.

For Hi-C sequencing, cells were cross-linked, digested with restriction enzymes, end-repaired, circularized, and purified to generate Hi-C libraries. The libraries were sequenced using an Illumina platform with PE150 reads. The raw data were filtered using SOAPnuke (v1.5.6) (with the same parameters as those used for short-read filtering) to remove adapters and low-quality reads (with >10% bases of quality ≤20 or >1% N bases), thereby retaining clean data for subsequent analysis.

Genome assembly was conducted using PacBio HiFi reads and assembled with hifiasm (v0.16.0) and purge_dups (v1.2.5), to generate contigs and deheterozygote sequences. This was followed by Hi-C sequencing data for chromosome-level scaffolding. To identify valid interactions, Hi-C reads were filtered using HiC-pro (v3.1.0) and aligned using Juicer (v1.5), whereafter they were processed using the 3d-dna (v180922) pipeline to cluster, order, and orient contigs into chromosome groups. This involved filtering small scaffolds, correcting misassemblies via interaction consistency analysis, and merging scaffolds using homology and long-range interaction patterns. Final manual validation with JuiceBox (v1.11.08), adjusting the contig order and orientation based on the interaction heatmaps. The assembly completeness was evaluated via BUSCOs (v5.2.2) with the eukaryota_odb10 dataset.

Detailed sequencing information is provided in Tables S2-S4, and the assembly statistics are shown in Table S5. The sequence interaction matrices for the Hi-C assembly are presented in Fig. S2. The statistical analysis of chromosome assemblies for *Physalia utriculus* (2n = 20) and *Porpita porpita* (2n = 14), with genomic loading rates of 92.28% and 96.83 %, respectively, is summarised in Table S6. A schematic representation of the genomic characteristics are shown in Fig. S3. Benchmarking Universal Single-Copy Orthologs (BUSCOs) assessment using the eukaryota_odb10 database revealed that our assemblies captured 95.68% and 96.07% of complete BUSCOs for *P. utriculus* and *P. porpita*, respectively,indicating that the assemblies had a high level of completeness and accuracy (Table S7).

**Genome annotation**

Repetitive elements were annotated using a combination of homology-based tools, such as RepeatMasker (v4.1.2) and RepBase (v21.12), and *de novo* prediction pipelines conducted using RepeatModeler (v2.0.2a) and TandemRepeatsFinder (v4.09). Protein-coding genes were annotated using a multi-strategy approach integrating homology-based prediction, *de novo* prediction, and transcriptome data. For *P. utriculus*, *Hydractinia symbiolongicarpus* (GCF_029227915.1), *Turritopsis rubra* [3], *Clytia hemisphaerica* (GCF_902728285.1), *Hydra vulgaris* (GCF_038396675.1), *Aurelia coerulea* [3], and *Rhopilema esculentum* [4] were used as reference species, and *P. utriculus* included as a reference species for *P. porpita*, with gene structures predicted via Exonerate (v2.2.0) by aligning homologous protein sequences to the genome. Augustus (v3.3.2), GlimmerHMM (v3.0.4), and Genscan (v1) were used for *de novo* prediction to identify open reading frames and splice sites based on statistically significant genomic features. The scRNA-seq data from the *P. utriculus* pneumatophore, tentacular palpons, gastrozooid, and gonodendron, and from the *P. porpita* mantle, dactylozooids, gastrozooid, and gonozooids, which were aligned to the genome with Hisat2 (v2.0.4), were reconstructed into transcripts using STRINGTIE (v2.1.3b) and further processed via TransDecoder (v3.0.1) and PASA (v2.5.2) to identify coding regions; publicly available RNA-seq data for different zooid life stages of *P. utriculus* were also incorporated [5]. EVidenceModeler (v2.0.0) was used to integrate these datasets to generate a non-redundant (NR) gene set, prioritizing gene structures supported by multiple lines of evidence. Gene functions were annotated using InterProScan (v5.62-94.0) for IPR and GO database annotation, followed by homologous structure searching against functional databases, including NR, Kyoto Encyclopedia of Genes and Genomes (KEGG), Swiss-Prot, KOG, TREMBL, and NOG, using the DIAMOND (v0.8.24.86) method.

The repetitive elements analysis is summarized in Table S8. A total of 29,087 and 34,914 genes were identified from *P. utriculus* and *P. porpita* genomes, respectively (Table S9), and 97.93% and 92.24% of the predicted genes were annotated using multiple databases (Table S10). Non-coding RNAs (ncRNAs) were identified via dedicated algorithms: ribosomal RNAs (rRNAs) were predicted using RNAmmer (v1.2), transfer RNAs (tRNAs) were identified via tRNAscan-SE (v2.0.12), small RNAs (sRNAs), microRNAs (miRNAs), and small nuclear RNAs (snRNAs) were annotated by aligning against the Rfam (v14.1) database with Infernal (v1.1.1) (Table S11). Gene annotation completeness was evaluated using BUSCO (eukaryota_odb10), and the results showed that our gene sets contained 87.8% and 91.0% of complete ortholog genes from *P. utriculus* and *P. porpita*, respectively (Table S12), demonstrating a high level of gene annotation completeness.

**Phylogenetic analyses**

Protein datasets were obtained from NCBI [*Acropora digitifera* (GCF_000222465.1), *Nematostella vectensis* (GCF_932526225.1), *H. vulgaris*, and *C. hemisphaerica*], MGU (Marine Genomics Unit; https://groups.oist.jp/mgu) (*M. virulenta*), or other sources [*T. rubra* [3], *T. dohrnii* [6], *Sanderia malayensis* [7], *Chrysaora quinquecirrha* [8], *A. coerulea* [3], *Cassiopea xamachana* [9], *R. esculentum* [4], and *Nemopilema nomurai* [10], as well as *P. utriculus* and *P. porpita* (present study)]. Gene families were clustered using OrthoFinder (v2.5.5), with the default settings for 15 cnidarians. Orthologues were identified, and a phylogenetic tree inferred using STAG (v1.0.0), which uses data from multi-copy gene families, resulting in high accuracy. The divergence times were estimated using MCMCTREE in PAML (v4.10.7), with the following parameters: clock = 2, model = 3, BDparas = 1 1 0. Fossil calibrations were based on previous studies, with a constraint of 240 million years ago set for the split between *Acropora* and *Nematostella*, corresponding to the earliest scleractinian corals in the fossil record. Additional calibrations were made in accordance with those described by Khalturin et al.[11].

**Expansion, contraction, and loss of gene families**

Expansion and contraction in gene families were analyzed using the CAFÉ tool (v5.1.0) with -p-k2 parameters based on a birth-and-death model. The P-values were adjusted using the Benjamini-Hochberg method with Python’s p.adjust function, with a significance threshold set at a false discovery rate < 0.05. For comparative analyses, GO and KEGG annotations for all proteins were derived using Eggnog (v5.0). Gene families with ≥ 90% of members sharing identical annotations were classified as single functional families and assigned a weighting value of 1. For families containing sequences with multiple functional annotations, weighting values were distributed among annotations proportionally to their occurrence frequency within the family, such that the sum of all weights per family equaled 1.

Genes that lacked homologues within the species-specific clade but had homologues in the closest sister lineage were considered to have been lost in this specific clade. Gene loss analysis was conducted using regular shell commands. Genes that were present in other species but absent in *P. utriculus* were also manually searched and confirmed using BLAST of the sequences in the compared genome FASTA files.

Gene expansion and contraction results were estimated for each branch of the phylogenetic tree. *P. utriculus* had 99 significantly expanded and 177 significantly contracted gene families (Dataset S1). The lost gene families identified through shell commands and manual searches are presented in Dataset S2. GO enrichment analysis of contracted and lost gene families in *P. utriculus* was conducted using the GOseq R package; the top 25 enriched categories for each class are presented in Fig. S4 (P < 0.05). Complete lists of the significantly enriched categories for lost and contracted gene families are presented in Datasets S3 and S4, respectively.

**Positive selected and rapidly evolving genes**

To identify PSGs and REGs in the *P. utriculus* lineage, 10 other species were selected (*C. hemisphaerica*, *P. porpita*, *T. dohrnii*, *T. rubra*, *M. virulenta*, *S. malayensis*, *A. coerulea*, *C. xamachana*, *N. nomurai*, and *R. esculentum*) as an outgroup. The coding sequences of one-to-one orthologous genes in all species were extracted from the corresponding protein sequences using Perl scripts [3]. Multiple sequence alignment was conducted for these coding sequences using Prank (v170427). The optimized branch-site and branch models in the CODEML program implemented in the PAML 4 package were used to identify PSGs and REGs, respectively. For the identification of PSGs, the likelihood ratio test (LRT) and Bayesian Empirical Bayes analysis were performed in alternative (model = 2; NSsites = 2; fix_omega = 0; omega = 0) and neutral (model = 2; NSsites = 2; fix_omega = 1; omega = 1) branch-site models. The chi-square test was used to calculate P-value based on the difference in likelihood values between these two models, assessing the model fit. Finally, the adjusted P-value was calculated using the Benjamini–Hochberg method to identify PSGs based on a threshold of P <0.05. For the identification of REGs, alternative (model = 2; NSsites = 0) and null (model = 0; NSsites = 0) branch models were used for LRT analysis. The chi-square test was also used to calculate the P-value. The criteria for identifying REGs were as follows: 1) adjusted P-value <0.05; 2) foreground dN/dS > background dN/dS; and 3) foreground dN/dS <3. These PSGs and REGs were functionally annotated according to the EggNOG (v5.0.2) (default parameters) and InterProScan databases.

A total of 113 PSGs and 91 REGs were identified in *P. utriculus* (adjusted *P*-value <0.05, Datasets S5 and S6).

**Single-cell RNA sequencing**

To characterize the cellular landscape of *P. utriculus*, scRNA-seq was conducted on complete colonies and pneumatophore samples that were collected separately from healthy adult *P. utriculus* colonies. As *P. utriculus* colonies are relatively large, we excised representative tissue fragments of approximately 3–5 mm from different regions (including the pneumatophore, tentacular palpons, gastrozooid, and gonodendron) of the colony and pooled them as whole-animal samples; for pneumatophore samples, approximately half of the pneumatophore tissue was excised for dissociation. Samples were rinsed twice in Ca²⁺- and Mg²⁺-free filtered artificial seawater (CMFASW; 25 g/L NaCl, 0.8 g/L KCl, and 0.04 g/L NaHCO₃, pH 8.5), minced into fragments using sterile scalpels, and immediately transferred to 2 mL of digestive buffer containing 3.6 mg/mL dispase II (Coolaber, Beijing, China) and 1.25 mg/mL collagenase III (ACMEC, Shanghai, China) in CMFASW. Digestion was performed at room temperature for 20 min with gentle pipetting every 5 min. The reaction was quenched by adding 8% (v/v) foetal bovine serum (FBS). The resulting cell suspension was centrifuged at 500 ×*g* for 5 min at 4 °C, resuspended in pre-chilled CMFASW, and filtered through a 40-μm cell strainer (FALCON; Corning, NY, USA) to obtain a single-cell suspension.

Cell viability was assessed using a low concentration of Calcein AM (2 μM) and Draq7™ (0.3  μM) for staining. Only cell suspensions with viability >90% were used for downstream single-cell capture. The BD Rhapsody™ system (BD Biosciences, Franklin Lakes, NJ, USA) was used for cell capture and the construction of single-cell RNA-seq libraries. The library quality was evaluated using a Qubit High Sensitivity DNA assay (Thermo Fisher Scientific, Waltham, MA, USA). Sequencing was performed using an Illumina NovaSeq X Plus platform (Illumina, San Diego, CA, USA) with 150-bp paired-end reads. The raw sequencing data were processed using the BD Rhapsody™ Sequence Analysis Pipeline (version 1.2) available from Seven Bridges (https://www.sevenbridges.com) following the manufacturer's protocol. Clean reads were aligned to the *P. utriculus* reference genome, and gene expression count matrices were generated accordingly. The sc-RNA sequencing information is provided in Table S13.

Unique molecular identifier (UMI) count matrices were imported into R (v 4.3.2) and processed using the Seurat package (v5.2.0) (https://satijalab.org/seurat/). To remove ambient RNA contamination and potential doublets, low-quality transcriptomes were filtered with the following criteria: (1) cells with <200 detected genes were excluded; (2) genes expressed in <3 cells were removed; and (3) cells with total UMI counts of <100 were discarded. These filters were applied independently to both the complete colony and pneumatophore datasets of *P. utriculus*. Following quality control, datasets from the two samples were merged and analysed using the standard Seurat workflow. Violin plots showing the number of UMIs and genes per cell of the complete colony and pneumatophore are presented in Fig. S7.

After quality control and clustering, 25 distinct cell populations were identified in the complete colony and 14 in the pneumatophore, each was characterised by clearly defined marker gene expression (Figs S8 and S13). Due to limitations in genome annotation, some clusters could not be classified with confidence. However, a subset of the cell types was annotated by integrating reference data from *A. coerulea* and orthologous gene information from other cnidarians such as *C. hemisphaerica* and *H. vulgaris*. We focused on identifying epidermal/muscle cells in *P. utriculus* (marked by *Myh6*, *Mys*, *Tpm1*, and *Mlck*) and neural cells (expressing *neuroglian*, *neurocalcin*, *opsin*, *Rfamide*, *OctB2R*, and *D(1C) dopamine receptor-like*).

**Cross-species comparative analysis**

The complete colony dataset of *P. utriculus* was compared with published single-cell transcriptomic datasets from *A. coerulea* (medusa stage) [3] and *H. vulgaris* [12] for cross-species comparisons. For cross-species integration of neural and epidermal/muscle cell populations, bidirectional transcriptome alignment between target species was performed using the SAMap tool to generate a matrix of homologous genes. Based on the alignment results, the following criteria were applied to define high-confidence homologous pairs: percent identity ≥30 and e-value ≤1e^-5^.

The alignment results were further filtered according to the following criteria: (1) the top five hits ranked by bit score in the target species were retained for each query transcript; (2) reciprocal best hits were identified by cross-matching the top dive results in both alignment directions; and (3) the mean bit score and percentage identity were calculated to represent the homologous pair in cases with multiple valid matches. The resulting homologous gene pairs were used to unify the gene expression matrices across species. Neural and epidermal/muscle cell populations were extracted from each dataset based on prior annotations, and only genes in the homologous set retained for downstream analysis.

The merged datasets were integrated in Seurat (v5.2.0) using the standard workflow. Batch effects across species were corrected using CCA via the FindIntegrationAnchors and IntegrateData functions. Subsequently, the integrated dataset was scaled and subjected to principal component analysis (PCA) and uniform manifold approximation and projection for dimensionality reduction. The DEGs among different samples or species were identified using the FindAllMarkers function in Seurat (v5.2.0), with a log2 fold change threshold set to 0.1 (log2fc.threshold = 0.1). The resulting DEGs were subsequently used for GO enrichment analysis based on the genome annotation information using ClusterProfiler [13].

**Homeodomain sequence analysis**

Candidate sequences, including the annotated *Hox* orthology sequences from *Homo sapiens*, *Branchiostoma floridae*, and *Drosophila melanogaster*, and the reported ANTP sequences of *S. malayensis* and *R. esculentum* were downloaded from GenBank and used to query the databases [7,14]. Protein and genomic sequences were queried using BLASTp (v2.2.30) and tBLASTn, respectively (using an e-value cutoff of 10e^-5^ and the -outfmt “6 sseqid sseq” flag to recover database hits). Each BLAST hit was vetted using the NR and Pfam-A databases; sequences were retained only if the program identified a homeodomain.

These sequences were aligned with MAFFT. The phylogenetic tree of *HoxL* proteins was constructed using the maximum likelihood (ML) method with the IQ-TREE software. The best-fit model, Q.insect+F+R7, was selected based on the Bayesian information criterion (BIC) and used with 1,000 bootstrap replicates. The phylogenetic tree of Hox1/lab orthologous genes was constructed with 1,000 bootstrap inferences followed by an ML search, with all free model parameters estimated using the JTT+G+I+F model in MEGA X64, where models with the lowest BIC scores were considered to describe the substitution pattern best. The classification and gene counts for cnidarian *HoxL* genes are provided in Dataset S12.

***Hox1* gene knockdown**

*Hox1* gene knockdown was performed using an RNA interference (RNAi) experiment. Two siRNAs (a mixture of three design sequences) targeting *Hox1* genes of *S. malayensis* and *A. coerulea* were designed by Genecefe Biotechnology (Jiangsu, China). The lyophilized siRNA was resuspended in RNase-free water to a stock concentration of 20 µM. Given that *S. malayensis* and *A. coerulea* continuously reside in seawater, the siRNA soaking transfection method was used for knockdown. Healthy *S. malayensis* and *A. coerulea* polyps of similar size and from the same rearing tank strain were seeded into a 48-well culture plate (one polyp per well in 500 μL of 30% filtered artificial sea water [FASW]). To investigate the function of *Hox1*, three treatment groups were established: control, negative control siRNA treatment, and si-*Hox1* treatment groups. Strobilation was induced using an induction buffer containing 50 µM 5-methoxy-2-methyl indole prepared in FASW.

For each group containing 48 polyps, all 48 wells were selected for the addition of transfection complex buffer. For the control group, 500 μL induction buffer was added per well. For the negative control treatment group, 50 μL of 1× riboFECTTM CP Buffer, 30 μL of 20 µM negative control siRNA, 5 μL riboFECTTM CP Reagent, and 415 μL induction buffer were mixed per well. For the si-*Hox1* groups, 50 μL of 1× riboFECTTM CP Buffer, 30 μL of 20 µM siRNA stock solution, 5 μL riboFECTTM CP Reagent, and 415 μL induction buffer were mixed per well. The transfection complex buffer was added to the respective treatment groups. All polyps were cultured at 20 °C for nine days, and the transfection complex replaced every three days. On day 6, polyps were collected for morphological observation, and two-thirds of the surviving polyps collected for RT-qPCR analysis. The remaining one-third remained in the RNAi experiment, with morphological observations made only on day 9 to determine whether the interfered groups could release ephyrae. The siRNA sequences used are listed in Table S14.

**Quantitative reverse transcription PCR**

On day 6 of the RNAi experiment, polyps were observed, and the number of individuals at each strobilation stage recorded. In each group, 32 surviving polyps were randomly selected for RT-qPCR. The mRNA samples were extracted using the Illustra Polyphenol Polysaccharide Plant Total RNA Extraction Kit (Zoman Biotechnology, Beijing, China), and cDNA synthesized using the One-Step gDNA Removal and cDNA Synthesis SuperMix Kit (TransGen Biotech, Beijing, China). The qPCR assay was performed using SYBR Green Master Mix (Thermo Fisher Scientific, Waltham, MA, USA) and an Applied Biosystems 7300 real-time PCR system (Thermo Fisher Scientific). All primers were diluted to 10 μM. Each analysis was repeated with four biological replicates. All samples were analyzed using *GAPDH* as a reference gene. Expression levels were calculated using the ΔΔCt method [15]. The data were analyzed using R software (v 4.5.1).

Laboratory cultured polyps of *S. malayensis* and *A. coerulea* were induced to strobilate using an induction buffer containing 50 µM 5-methoxy-2-methyl indole prepared in FASW. Cultured polyps and newly released ephyrae were collected and used for RT-qPCR to measure *Hox1* expression in the polyp and ephyra stages of *S. malayensis* and *A. coerulea*. Each analysis was repeated with five biological replicates. The data was analysed by R software (v 4.5.1). The RT-qPCR primer sequences used for the selected genes are presented in Table S16.

**Horizontal gene transfer**

Whether the heme oxygenase gene from *P. utriculus* had been horizontally acquired was evaluated using a two-step workflow [16]. A BLASTp search was conducted against the NR protein database with an e-value cutoff of 1e^−10^. The taxonomic information for each BLAST hit was assigned to three lineages: scyphozoa (recipient), animalia (excluding scyphozoa), and non-animal taxa (outgroup). The AI value and percentage of outgroup species (out_pct) were calculated. HGT determination was performed via phylogenetic analysis. Sequences were aligned using MAFFT, and ambiguously aligned regions trimmed using trimAl (v1.5.rev0). An ML tree was constructed using IQ-TREE with 1,000 ultrafast bootstrapping replicates. The resulting tree was visualized with ChiPlot (https://www.chiplot.online/).

**Metabolomic analysis.**

Tentacular palpons (n = 6) were excised from a healthy adult *P. utriculus* colony and gently rinsed with FASW to remove surface impurities, followed by rapid freezing in liquid nitrogen and lyophilisation in a freeze-dryer. Lyophilised samples (50 mg) were weighed and mixed with 1000 μL of extraction solution containing internal standards (methanol:acetonitrile:water = 2:2:1, with internal standard concentration of 20 mg/L), vortexed for 30 s, and homogenised with steel beads using a 45-Hz grinder for 10 min, followed by ice-water bath sonication for 10 min. Samples were incubated at -20 °C for 1 h, centrifuged at 12,000 rpm for 15 min at 4 °C, and 500 μL of the supernatant was transferred to an Eppendorf tube. The extract was dried in a vacuum concentrator, reconstituted with 160 μL of extraction solution (acetonitrile:water = 1:1), vortexed for 30 s, sonicated in an ice-water bath for 10 min, centrifuged again under the same conditions, and 120 μL of supernatant was transferred to a 2-mL injection vial. A quality control sample was prepared by pooling 10 μL from each sample for instrumental analysis.

The liquid chromatography–mass spectrometry (LC/MS) system for metabolomics analysis consisted of a Waters Acquity I-Class PLUS ultra-high-performance liquid chromatography tandem with a Waters Xevo G2-XS QTOF high-resolution mass spectrometer (operated by Biomarker Technologies Co., Ltd., Beijing, China). Chromatographic separation was achieved on a Waters Acquity UPLC HSS T3 column (1.8 μm, 2.1 × 100 mm) using a binary gradient of 0.1% formic acid in water (mobile phase A) and 0.1% formic acid in acetonitrile (mobile phase B) for both positive and negative ionisation modes. Samples were injected at a volume of 1 μL. The Waters Xevo G2-XS QTOF high-resolution mass spectrometer can collect primary and secondary mass spectrometry data in MSe mode under the control of the acquisition software (MassLynx V4.2, Waters). Dual-channel data acquisition can be simultaneously performed in each data acquisition cycle at both low and high collision energies. The low collision energy is 2V, the high collision energy range is 10–40 V, and the scanning frequency is 0.2 s for a mass spectrum analysis. The parameters of the ESI ion source were as follows: capillary voltage, 2000 V (positive ion mode) or −1500 V (negative ion mode); cone voltage, 30 V; ion source temperature, 150 ℃; desolvent gas temperature, 500 ℃; backflush gas flow rate, 50 L/h; and desolvatising gas flow rate, 800 L/h.

The raw data collected using MassLynx (v4.2) were processed using Progenesis QI software for peak extraction, peak alignment, and other data processing operations, based on the online METLIN database and Biomark’s self-built library for identification. Theoretical fragment identification was performed, with the mass deviation of parent ions within 100 ppm and that of fragment ions within 50 ppm. The original peak area information was normalized with the total peak area, and a follow-up analysis then performed. PCA and Spearman correlation analysis were used to judge repeatability of the samples within the group and the QC samples. The KEGG, HMDB, and LIPID MAPS databases were searched for classification and pathway information. A total of 28,381 peaks were detected, from which 4,268 metabolites were annotated (Dataset S13).

**Proteomic analysis**

Tentacular palpons (n = 6) were also excised from a healthy *P. utriculus* adult colony and gently rinsed with FASW to remove surface impurities. One palpon was fixed in 4% paraformaldehyde overnight and observed under an optical microscope to examine nematocyst morphology.

Five palpons were frozen in liquid nitrogen and stored at −80 ℃ for proteomic analysis performed by Biomarker Technologies Co., Ltd. Urea (300 μL, 8 M) was added to each sample, and 10% protease inhibitor was added to the lysate. After centrifuging at 14,100 ×*g* for 20 min, the supernatant was collected. The protein concentration was determined using the Bradford method, and the remaining samples were frozen at −80 ℃.

A 50 µg aliquot of extracted proteins from each sample was reduced. A 200 mM dithiothreitol (DTT) solution was added, and the samples were incubated at 37 °C for 1 h. Each sample was diluted eight times by adding 50 mM ammonium bicarbonate (ABC) buffer. Trypsin (trypsin: protein = 1:25) was added, and the samples were incubated at 37 ℃ overnight. The following day, digestion was terminated by adding 50 μL of 0.1% formic acid (FA). The C18 column was pre-washed with 100 μL of 100% acetonitrile (ACN) (centrifuged at 1200 rpm for 3 min), followed by a single wash with 100 μL of 0.1% FA (same centrifugation conditions). After the collection tube was replaced, ≤30 μg of sample was applied and centrifuged. The column was then washed twice with 100 μL of 0.1% FA and once with 100 μL of pH 10 water. Elution was performed using a 10-step gradient (6%, 9%, 12%, 15%, 18%, 21%, 25%, 30%, 35%, and 50% ACN in pH 10 water). The eluates were pooled into three fractions: 6%, 15%, 25%, 50%; 9%, 18%, 30%; and 12%, 21%, 35%. Fractions were lyophilised and stored at −80 °C until liquid chromatography with tandem mass spectrometry (LC–MS/MS) analysis.

The lyophilised fraction was reconstituted with 20 µL of 2% methanol and 0.1% formic acid. Samples were centrifuged at 12,000 rpm for 10 min, and the supernatants were aspirated to load the samples. The loading volume was 10 μL, and the sandwich loading method was used. The loading pump flow rate was 300 nL/min for 15 min. The separation flow rate was maintained at 300 nL/min for 120 min. Mobile phase A contained 0.1% formic acid in water, whereas mobile phase B contained 0.1% formic acid in ACN. The LC gradient was as follows: Solvent B was maintained at 4% for the first 8 min, increased from 4% to 10% over 3 min, 10–25% over 77 min, 25–50% over 10 min, 50–99% over 4 min, maintained at 99% for 6 min, decreased from 99% to 4% over 4 min, and then maintained at 4% for 8 min.

Label-free mass spectrometry was performed using a Thermo Orbitrap Fusion mass spectrometer. The scan events were set as a full MS scan of 250–1450 m/z at a mass resolution of 120,000, followed by a CID MS/MS scan with an isolation window repeated on the 20 most intense ions selected from the previous full MS scan. The normalised collision energy was set to 30%, and the activation time was set to 50 ms. The second stage used linear ion trap fast mode for data acquisition, with an automatic gain control (AGC) setting of 7000; a maximum injection time of 35 ms, and a dynamic exclusion time of 18 s. The resulting MS/MS data were processed using Maxquant 1.5.2.8.

Identification was set as follows: precursor ion mass tolerance, ± 15 ppm; fragment ion mass tolerance, ± 0.5 Da; maximum missed cleavages, 2; static modification, carboxyamidomethylation (57.021 Da) of Cys residues; dynamic modification, oxidation modification (+15.995 Da) of Met residues. A total of 746 proteins were detected.

Putative toxins were identified in the predicted proteome using BLASTp. Protein sequences were annotated for sequence homology against manually curated venom proteins and toxins in the UniProtKB/Swiss-Prot database (UniProtKB 2025_04) with an e-value threshold of 1e^-6^. Following annotation, hits were filtered and classified: only sequences with e-values ≤1e^-20^ and matching lengths of >10 amino acids were shortlisted as candidate toxins. Genes corresponding to candidate toxin proteins were further queried for annotation; those annotated as previously reported toxin proteins or containing toxic domains were designated as confirmed toxins. Toxin protein files are provided in Dataset S10.

**Synteny analysis of the *CtrA* gene families**

The *CtrA* genes in *P. utriculus* and other species (*P. porpita*, *T. rubra*, *H. vulgaris*, *S. malayensis*, *R. esculentum*, *A. coerulea*, and *N. vectensis*) were identified using nr annotation and sorted for genomic contig/scaffold/linkage. The synteny relationships were determined using ChiPlot. The protein structure prediction of *CtrA* genes was performed using AlphaFold 3 [17]. An ML tree was generated with IQ-TREE using protein sequences for these species. Bootstrap support was established with 1,000 iterations for Ultra-Fast Bootstrapping.

**Statistics and reproducibility**

In the RNAi experiment, 48 polyps of each group were collected for morphological observation. In the RT-qPCR experiment, each analysis was repeated with at least four biological replicates. Six tentacular palpons and five tentacular palpons were used for toxin metabolomic and proteomic analysis, respectively. No statistical method was used to predetermine sample size. No data were excluded from the analyses.

**Figure S1.**

Morphological photographs of (**a**) *Physalia utriculus* and (**b**) *Porpita porpita*. (**c**). Phylogenetic analysis of 138 mitochondrial genomes shows reciprocal monophyly of lineages. (d). Phylogeny of 118 16S ribosomal RNA sequences, assembled using in silico PCR from reads, and publicly available sequences from NCBI.

**Figure S2.**

Hi-C contact maps of (**a**) *Physalia utriculus* and (**b**) *Porpita porpita* genome assemblies. Chromosome-level genome assemblies comprising 10 and 7 chromosome-level scaffolds.

**Figure S3.**

Schematic representation of the genomic characteristics of (**a**) *Physalia utriculus* and (**b**) *Porpita porpita*. Track a: Protein-coding genes on the plus strand. Track b: Protein-coding genes on the minus strand. Track c: Gene density distribution with a sliding window of 1 Mb. Darker red indicates a higher density. Track d: Coverage ratio of repeat sequences in each 1 Mb genomic region. Track e: GC content percentage of each 1 Mb sliding window. Track f: Schematic presentation of major interchromosomal relationships.

**Figure S4.**

Enriched Gene Ontology (GO) functional categories of the contracted and lost gene families in the *Physalia utriculus* genome.

**Figure S5.**

Number of expanded, contracted, and lost gene families related to development, toxins, the neuromuscular system, and a sessile lifestyle in cnidarians, with potential associations with medusa loss, toxicity, and floating behavior in *Physalia utriculus*.

**Figure S6.**

Overview of the genomic determinants underlying the morphological characteristics of *Physalia utriculus*. Expanded genes (red), contracted/lost genes (grey), positively selected genes (PSGs, orange), and rapidly evolving genes (REGs, green), are associated with processes such as gas production, sessile polyp development, anatomical structure formation, nervous system development, and muscle assembly and contraction, and play pivotal roles in shaping the distinctive morphological features such as obligate habits and loss of medusa in *P. utriculus*.

**Figure S7.**

Violin plots indicating the number of unique molecular identifiers (UMIs) and *Physalia utriculus* genes per cell of the (**a**) complete colony and (**b**) pneumatophore.

**Figure S8.**

*Physalia utriculus* complete colony cell atlas. (**a**) Uniform manifold approximation and projection (UMAP) visualisation of cell clusters in the complete *P. utriculus* colony, colored according to Louvain clustering identity and annotated based on marker genes. (**b**) Dot plot showing the expression of the selected marker genes per cell type. Dot sizes indicate percentages of cells within a cell type in which a given marker is detected; dot color intensities indicate average expression levels.

**Figure S9.**

Feature plots depicting the expression levels of genes that are either contracted or lost in the neural cells of *Physalia utriculus* across three distinct jellyfish species. The genes were detected in the neural cells of *Aurelia coerulea* but not in the neural cells of *Hydra vulgaris* or *P. utriculus*.

**Figure S10.**

Feature plots showing the expression levels of positively selected genes (PSGs) and lost genes of *Physalia utriculus* in epidermal/muscle cells across three jellyfish species. Genes were detected in the epidermal/muscle cells of *Hydra vulgaris* and *P. utriculus* but showed no expression in the epidermal/muscle cells of *Aurelia coerulea*.

**Figure S11**.

Cnidarian ontogeny and *Hox1* gene evolution. (**a**) Schematic of cnidarian ontogeny from the benthic sessile to floating to free-swimming stages. (**b**) Phylogenetic tree of *Hox1* homologous genes from various species.

**Figure S12.**

Conserved sequences and predicted protein structures of *Hox1* homologues in cnidarians. (**a**) Multiple sequence alignment of conserved homeodomain regions in *Hox1* homologues across cnidarians reveals high conservation. (**b**) Schematic comparison of conserved sequence motifs and predicted protein structures of Hox1 homologues in cnidarians with distinct ontogenetic traits. *Hox1* in jellyfish (possessing medusae), *Cnox1* in *Hydra* and *Ax6* in anthozoans (lacking medusae) show divergent sequence features. These sequence differences correlate with predicted structural variations in the homeodomain-containing proteins, potentially underlying functional disparities related to medusa formation and other ontogenetic-associated processes.

**Figure S13.**

*Physalia utriculus* pneumatophore cell atlas. (**a**) Uniform manifold approximation and projection (UMAP) visualisation of cell clusters in the pneumatophore of *P. utriculus*, colored based on Louvain clustering identity and annotated based on marker genes. (**b**) Dot plot showing the expression of the selected marker genes per cell type. Dot sizes indicate the percentages of cells within a cell type in which a given marker is detected; dot color intensities indicate average expression levels.

**Figure S14.**

Feature plots of toxin protein-coding genes in the complete *Physalia utriculus* colony cell atlas. Genes related to toxin proteins are specifically or highly expressed in cluster 24, which may be associated with the production of toxins or venom in *P. utriculus*. Members of the expanded gene CTG family are highlighted in red.

**Figure S15.**

(**a**) Feature plots of the Hox1 gene in the medusa of *Turritopsis rubra* from the cell atlas. (**b**) Uniform manifold approximation and projection (UMAP) visualisation of the merged dataset across different stages of *T. rubra*, data were obtained from Dong *et al*.[3].

Table S1.**Samples used for genome and Hi-C sequencing.**

| Species Sampling | Origin | Sampling date | Genome sequencing | Hi-C sequencing |
| --- | --- | --- | --- | --- |
| *P. utriculus* | Zhaoshu Island, Hainan, China | 2024.1.26 | pneumatophore | tentacular palpons |
| *P. porpita* | Zhaoshu Island, Hainan, China | 2024.1.30 | colony (complete) | colony (complete) |

Table S2. **Genome sequencing data by Illumina.**

| Species | Read number | Total base (bp) | Depth | GC (%) | Q20 (%) | Q30 (%) |
| --- | --- | --- | --- | --- | --- | --- |
| *P. utriculus* | 1,407,975,474 | 211,196,321,100 | 57× | 33.59 | 99.42 | 97.92 |
| *P. porpita* | 342,859,678 | 51,428,951,700 | 11× | 30.49 | 99.46 | 98.06 |

Table S3. **Genome sequencing data by PacBio.**

| Species | Number | Total bases (bp) | Depth | Mean length (bp) | N50 (bp) | N90 (bp) | Max length (bp) | Min length (bp) |
| --- | --- | --- | --- | --- | --- | --- | --- | --- |
| *P. utriculus* | 8,587,943 | 148,691,298,483 | 42× | 17,313 | 17,188 | 14,299 | 65,793 | 1,001 |
| *P. porpita* | 12,032,399 | 191,636,398,636 | 42× | 15,926 | 15,871 | 13,258 | 62,305 | 1,003 |

Table S4. **Sequencing data evaluation for Hi-C sequencing.**

| Species | Read number | Total base (bp) | Depth | GC (%) | Q20 (%) | Q30 (%) |
| --- | --- | --- | --- | --- | --- | --- |
| *P. utriculus* | 2,280,350,286 | 342,052,542,900 | 86× | 37.84 | 97.97 | 94.97 |
| *P. porpita* | 2,020,999,772 | 303,149,965,800 | 67× | 35.75 | 95.62 | 90.75 |

Table S5. **Summary of genome sizes and assembly statistics.**

| Species | Assembly size (bp) | Scaffold number | Scaffold N50(bp) | Contig number | Contig N50 (bp) | GC (%) | Load rate (%) |
| --- | --- | --- | --- | --- | --- | --- | --- |
| *P. utriculus* | 3,486,154,839 | 1,589 | 326,157,332 | 4,511 | 1,896,632 | 31.31 | 92.28 |
| *P. porpita* | 4,527,808,484 | 291 | 623,494,391 | 3,218 | 3,780,284 | 29.52 | 96.83 |

Table S6. **Statistics of the anchored chromosomes for genomes of *Physalia utriculus* and *Porpita porpita*.**

| Species | Chromosome | Length | Species | Chromosome | Length |
| --- | --- | --- | --- | --- | --- |
| *P. utriculus* | Chr01 | 476,270,338 | *P. porpita* | Chr01 | 678,702,333 |
|  | Chr02 | 467,093,843 |  | Chr02 | 654,731,283 |
|  | Chr03 | 447,668,126 |  | Chr03 | 632,621,813 |
|  | Chr04 | 336,221,541 |  | Chr04 | 623,494,391 |
|  | Chr05 | 326,157,332 |  | Chr05 | 620,656,412 |
|  | Chr06 | 263,124,829 |  | Chr06 | 598,550,703 |
|  | Chr07 | 259,506,744 |  | Chr07 | 575,630,987 |
|  | Chr08 | 229,454,017 |  | Total unChr | 143,420,562 |
|  | Chr09 | 218,148,505 |  | Total length | 4,527,808,484 |
|  | Chr10 | 193,702,441 |  |  |  |
|  | Total unChr | 269,099,323 |  |  |  |
|  | Total length | 3,486,447,039 |  |  |  |

Table S7. **Evaluation of genome sequences completeness and coverage.**

| Species | Complete BUSCOs (%) | Complete and single-copy BUSCOs (%) | Complete and duplicated BUSCOs (%) | Fragmented BUSCOs (%) | Missing BUSCOs (%) | Mapping rate  (%) | Coverage (%) |
| --- | --- | --- | --- | --- | --- | --- | --- |
| *P. utriculus* | 95.68 | 85.09 | 10.58 | 3.52 | 0.78 | 99.92 | 92.60 |
| *P. porpita* | 96.07 | 77.25 | 18.82 | 2.35 | 1.56 | 99.63 | 96.93 |

Table S8. **Statistics of transposable elements (TEs) in *Physalia utriculus* and *Porpita porpita* genomes.**

| Species | Type | Repbase TEs | | ProteinMask TEs | | Combined TEs | |
| --- | --- | --- | --- | --- | --- | --- | --- |
|  |  | Length (bp) | Rate (%) | Length (bp) | Rate (%) | Length (bp) | Rate (%) |
| *P. utriculus* | DNA | 1,337,172 | 0.0384 | 66,063,333 | 1.8949 | 67,070,127 | 1.9237 |
|  | LINE | 23,739,737 | 0.6809 | 521,272,905 | 14.9514 | 522,507,591 | 14.9868 |
|  | LTR | 339,581 | 0.0097 | 29,637,139 | 0.8501 | 29,893,730 | 0.8574 |
|  | SINE | 2,475,740 | 0.0710 | 0 | 0.0000 | 2,475,740 | 0.0710 |
|  | Other | 393,973,240 | 11.3001 | 4,092 | 0.0001 | 4,092 | 0.0001 |
|  | Unknown | 11,984,189 | 0.3437 | 0 | 0.0000 | 11,984,189 | 0.3437 |
|  | Total | 433,762,275 | 12.4414 | 616,940,895 | 17.6954 | 1,026,154,259 | 29.4327 |
| *P. porpita* | DNA | 422,448 | 0.0093 | 18,373,334 | 0.4058 | 18,697,235 | 0.4129 |
|  | LINE | 24,522,741 | 0.5416 | 532,611,742 | 11.7631 | 534,623,916 | 11.8076 |
|  | LTR | 525,488 | 0.0116 | 46,904,422 | 1.0359 | 47,108,830 | 1.0404 |
|  | SINE | 670,037 | 0.0148 | 0 | 0.0000 | 670,037 | 0.0148 |
|  | Other | 519,165,960 | 11.4662 | 26,861 | 0.0006 | 0 | 0.0000 |
|  | Unknown | 67,033 | 0.0015 | 684 | 0.0000 | 67,717 | 0.0015 |
|  | Total | 545,112,163 | 12.0392 | 597,877,955 | 13.2046 | 1,118,454,009 | 24.7019 |

Table S9. **Statistics of the gene prediction in *Physalia utriculus* and *Porpita porpita*.**

| Species | Gene set | Number | Average gene length (bp) | Average CDS length (bp) | Average exon per gene | Average exon length (bp) | Average intron length (bp) |
| --- | --- | --- | --- | --- | --- | --- | --- |
| *P. utriculus* | EVM | 29,087 | 10,791.34 | 1,753.89 | 2.74 | 639.31 | 5,177.72 |
| *P. porpita* | EVM | 34,914 | 22,637.15 | 1,470.53 | 3.35 | 438.78 | 9,000.96 |

Table S10. **Statistics of functional annotation of protein-coding genes in *Physalia utriculus* and *Porpita porpita*.**

| Species | Annotation database | Number | Percent (%) |
| --- | --- | --- | --- |
| *P. utriculus* | TREMBL | 28,284 | 97.24 |
|  | IPR | 18,337 | 63.04 |
|  | NOG | 9,851 | 33.87 |
|  | SWISSPROT | 3,562 | 12.25 |
|  | GO | 8,958 | 30.80 |
|  | KEGG | 4,430 | 15.23 |
|  | NR | 15,574 | 53.54 |
|  | KOG | 3,477 | 11.95 |
|  | Annotated | 28,484 | 97.93 |
|  | Unannotated | 603 | 2.07 |
| *P. porpita* | TREMBL | 31,555 | 90.38 |
|  | IPR | 24,118 | 69.08 |
|  | NOG | 27,519 | 78.82 |
|  | SWISSPROT | 19,866 | 56.90 |
|  | GO | 14,592 | 41.79 |
|  | KEGG | 7,130 | 20.42 |
|  | NR | 31,277 | 89.58 |
|  | KOG | 17,418 | 49.89 |
|  | Annotated | 32,205 | 92.24 |
|  | Unannotated | 2,709 | 7.76 |

Table S11. **Statistics of the non-coding RNA of *Physalia utriculus* and *Porpita porpita* genomes.**

| Species | Type | Copy | Average length (bp) | Total length (bp) | % in genome |
| --- | --- | --- | --- | --- | --- |
| *P. utriculus* | tRNA | 50,801 | 75.26 | 3,823,738 | 0.1097 |
|  | rRNA | 392 | 637.71 | 249,985 | 0.0072 |
|  | sRNA | 9,854 | 50.47 | 497,424 | 0.0143 |
|  | snRNA | 6,038 | 62.13 | 375,183 | 0.0108 |
|  | miRNA | 445,986 | 28.48 | 12,704,490 | 0.3644 |
| *P. porpita* | tRNA | 43,090 | 74.45 | 3,208,449 | 0.0709 |
|  | rRNA | 1,225 | 1,200.74 | 1,470,918 | 0.0325 |
|  | sRNA | 5,116 | 54.83 | 280,512 | 0.0062 |
|  | snRNA | 4,913 | 56.23 | 276,273 | 0.0061 |
|  | miRNA | 464,893 | 29.82 | 13,867,354 | 0.3063 |

Table S12. **Benchmarking universal single-copy orthologs (BUSCO) evaluation of annotated results.**

| n=255 | Percent (%) | |
| --- | --- | --- |
|  | *P. utriculus* | *P. porpita* |
| Complete BUSCOs | 87.8 | 91.0 |
| Complete and single-copy BUSCOs | 81.2 | 77.6 |
| Complete and duplicated BUSCOs | 6.7 | 13.3 |
| Fragmented BUSCOs | 5.1 | 7.5 |
| Missing BUSCOs | 7.1 | 1.6 |

Table S13. **The single-cell RNA (scRNA) sequencing information of *Physalia utriculus*.**

| Sample | Platform | Data (Gb) | Reads (bp) | Mapping rate (%) | Selected cells | Detected genes |
| --- | --- | --- | --- | --- | --- | --- |
| Complete colony | BD Rhapsody | 83.59 | 513,346,811 | 97.04 | 14,339 | 9,359 |
| Pneumato-  phore | BD Rhapsody | 74.80 | 448,769,382 | 96.03 | 9,554 | 9,405 |

Table S14. **Sequences of the small interfering RNA (siRNA) used in the study.**

| Species | Number | Sequence (5'-3') |
| --- | --- | --- |
| *S. malayensis* | 1 | mCmAmUmAmGmUmUmCmAmUmUmGmAmCmGmAmAmUmATT |
|  | 2 | mGmCmAmAmCmGmUmAmGmAmUmAmAmGmAmCmAmAmATT |
|  | 3 | mGmCmAmAmCmAmAmCmAmAmCmAmAmCmAmAmGmAmATT |
| *A. coerulea* | 1 | mCmCmUmAmUmGmGmAmCmUmAmCmCmAmAmGmAmUmATT |
|  | 2 | mCmCmAmGmAmGmAmAmGmCmAmGmAmCmUmCmCmUmATT |
|  | 3 | mGmCmAmCmCmUmCmUmCmAmGmAmAmUmCmAmCmAmATT |
| Negative Control | 1 | UUCUCCGAACGUGUCACGUTT |

Table S15. The number of polyps under three stages of each groups**.**

| Time | Species | Group | ES | AS | EP | Total |
| --- | --- | --- | --- | --- | --- | --- |
| Day 6 | *S. malayensis* | C | 4 | 44 | 0 | 48 |
|  |  | NC | 9 | 39 | 0 | 48 |
|  |  | Si-*Hox1* | 42 | 6 | 0 | 48 |
|  | *A.coerulea* | C | 0 | 48 | 0 | 48 |
|  |  | NC | 0 | 48 | 0 | 48 |
|  |  | Si-*Hox1* | 48 | 0 | 0 | 48 |
| Day 9 | *S. malayensis* | C | 0 | 1 | 15 | 16 |
|  |  | NC | 0 | 3 | 13 | 16 |
|  |  | Si-*Hox1* | 14 | 2 | 0 | 16 |
|  | *A.coerulea* | C | 0 | 0 | 16 | 16 |
|  |  | NC | 0 | 1 | 15 | 16 |
|  |  | Si-*Hox1* | 10 | 6 | 0 | 16 |

Table S16. **Sequences of the RT-qPCR primer sequences of selected genes.**

| Gene | Sequence (5'-3') |
| --- | --- |
| Sma-Hox1 | F: GAAATACAAGCGTGAGCAGAAG |
|  | R: GGCCAACTGGGACTGTAACTAT |
| Sma-paramyosin | F: AGGCTGGCACTTGAGATTCC |
|  | R: CTGCCACGTCAGCAAGTTTC |
| Sma-Chrng | F: GCCTCGATTCCAACAGACCA |
|  | R: CAGAAGAGGGTGCAGCTTGA |
| Sma-Grm3 | F: CAAAGGCAATGGGATGGTGC |
|  | R: AGGATGGCAAGAAGACAGCC |
| Sma-GAPDH | F: ATTCCAGCCTCTACTGGTGC |
|  | R: AAACATCGGGTACTGGCACA |
| Aco-Hox1 | F: CGAAATACCAGAGAAGCAGACTC |
|  | R: CTCTTTTCTTGTTGGCTCCAGT |
| Aco-paramyosin | F: CGGCGACATGAAAGTTGAGC |
|  | R: GCTGGCGTCTTCGACATTTG |
| Aco-Chrng | F: GCAAACGTTAGGCCTTCGAC |
|  | R: ACCCAGACCATCATGACACG |
| Aco-Grm3 | F: GCTGCTCAAATCTGCAGGACC |
|  | R: GCAATTAAACTGCTGCTCCCAG |
| Aco-GAPDH | F: GTCTGCCGATGCTCCAATGT |
|  | R: GGCTAAGGGTGCAAGGCAAT |

**Dataset S1.** The list of expanded/contracted gene families in selected cnidarians (Q-value<0.05).

**Dataset S2.** The list of lost genes in *P. utriculus* (P-value<0.05).

**Dataset S3.** GO enrichment of lost gene families in *P. utriculus* (P-value<0.05).

**Dataset S4.** GO enrichment of contracted gene families in *P. utriculus* (P-value<0.05).

**Dataset S5.** Positive selected genes (PSGs) identified in *P. utriculus* (P-value<0.05).

**Dataset S6.** Rapidly evolving genes (REGs) identified in *P. utriculus* (P-value<0.05).

**Dataset S7.** The different expressed genes (DEGs) of cross-species comparative analysis.

**Dataset S8.** GO enrichment of the different expressed genes (DEGs) from cross-species comparative analysis (P-value<0.05).

**Dataset S9.** Identification of toxicity-related metabolites in the tentacular palpons of *P. utriculus*.

**Dataset S10.** Identification of toxicity-related proteins in the tentacular palpons of *P. utriculus*.

**Dataset S11.** GO enrichment analysis of cluster 24 (P-value<0.05).

**Dataset S12.** Classification and gene counts for selected cnidarians *HoxL* genes.

**Dataset S13.** List of all metabolites dedicated in the tentacular palpons of *P. utriculus*.

**References**

1. Church SH, Abedon RB, Ahuja N *et al*. Population genomics of a sailing siphonophore reveals genetic structure in the open ocean. *Curr Biol* 2025; **35**: 3556-69.e6.
2. Allen GC, Flores-Vergara MA, Krasynanski S *et al*. A modified protocol for rapid DNA isolation from plant tissues using cetyltrimethylammonium bromide. *Nat Protoc* 2006; **1**: 2320-5.
3. Dong Z, Wang F, Liu Y *et al*. Genomic and single-cell analyses reveal genetic signatures of swimming pattern and diapause strategy in jellyfish. *Nat Commun* 2024; **15**: 5936.
4. Li Y, Gao L, Pan Y *et al*. Chromosome-level reference genome of the jellyfish *Rhopilema esculentum*. *GigaScience* 2020; **9**: giaa036.
5. Munro C, Zapata F, Howison M, et al. Evolution of gene expression across species and specialized zooids in Siphonophora. *Mol Biol Evol* 2022; **39**: msac027.
6. Hasegawa Y, Watanabe T, Otsuka R *et al*. Genome assembly and transcriptomic analyses of the repeatedly rejuvenating jellyfish *Turritopsis dohrnii*. *DNA Res* 2023; **30**: dsac047.
7. Nong W, Cao J, Li Y *et al*. Jellyfish genomes reveal distinct homeobox gene clusters and conservation of small RNA processing. *Nat Commun* 2020; **11**: 3051.
8. Xia W, Li H, Cheng W *et al*. High-quality genome assembly of *Chrysaora quinquecirrha* provides insights into the adaptive evolution of jellyfish. *Front Genet* 2020; **11**: 535.
9. Ohdera A, Ames CL, Dikow RB *et al*. Box, stalked, and upside-down? Draft genomes from diverse jellyfish (Cnidaria, Acraspeda) lineages: *Alatina alata* (Cubozoa), *Calvadosia cruxmelitensis* (Staurozoa), and *Cassiopea xamachana* (Scyphozoa). *GigaScience* 2019; **8**: giz069.
10. Kim HM, Weber JA, Lee N *et al*. The genome of the giant *Nomura*’s jellyfish sheds light on the early evolution of active predation. *BMC Biol* 2019; **17**: 1-12.
11. Khalturin K, Shinzato C, Khalturina M *et al*. Medusozoan genomes inform the evolution of the jellyfish body plan. *Nat Ecol Evol* 2019; **3**: 811-22.
12. Siebert S, Farrell JA, Cazet JF *et al*. Stem cell differentiation trajectories in *Hydra* resolved at single-cell resolution. *Science* 2019; **365**: eaav9314.
13. Yu G, Wang L, Han Y *et al*. ClusterProfiler: an R package for comparing biological themes among gene clusters. *Omics* 2012; **16**: 284-287.
14. Schiemann SM, Martín-Durán JM, Børve A *et al*. Clustered brachiopod *Hox* genes are not expressed collinearly and are associated with lophotrochozoan novelties. *Proc Natl Acad Sci U S A* 2017; **114**: E1913-22.
15. Rao X, Huang X, Zhou Z *et al*. An improvement of the 2ˆ(-delta delta CT) method for quantitative real-time polymerase chain reaction data analysis. *Biostat Bioinforma Biomath* 2013; **3**: 71-85.
16. Shen XX, Opulente DA, Kominek J *et al*. Tempo and mode of genome evolution in the budding yeast subphylum. *Cell* 2018; **175**: 1533-45.e20.
17. Abramson J, Adler J, Dunger J *et al*. Accurate structure prediction of biomolecular interactions with AlphaFold 3. *Nature* 2024; **630**: 493-500.
